# Glucose controls co-translation of structurally related mRNAs *via* the mTOR and eIF2 pathways in human pancreatic beta cells

**DOI:** 10.3389/fendo.2022.949097

**Published:** 2022-08-05

**Authors:** Manuel Bulfoni, Costas Bouyioukos, Albatoul Zakaria, Fabienne Nigon, Roberta Rapone, Laurence Del Maestro, Slimane Ait-Si-Ali, Raphaël Scharfmann, Bertrand Cosson

**Affiliations:** ^1^ Université Paris Cité, CNRS, Epigenetics and Cell Fate, Paris, France; ^2^ Université Paris Cité, Institut Cochin, INSERM, CNRS, Paris, France

**Keywords:** response to glucose, mRNA feature, translation regulation, Human beta cells, RNA co-regulation, mTOR, eIF2

## Abstract

Pancreatic beta cell response to glucose is critical for the maintenance of normoglycemia. A strong transcriptional response was classically described in rodent models but, interestingly, not in human cells. In this study, we exposed human pancreatic beta cells to an increased concentration of glucose and analysed at a global level the mRNAs steady state levels and their translationalability. Polysome profiling analysis showed an early acute increase in protein synthesis and a specific translation regulation of more than 400 mRNAs, independently of their transcriptional regulation. We clustered the co-regulated mRNAs according to their behaviour in translation in response to glucose and discovered common structural and sequence mRNA features. Among them mTOR- and eIF2-sensitive elements have a predominant role to increase mostly the translation of mRNAs encoding for proteins of the translational machinery. Furthermore, we show that mTOR and eIF2α pathways are independently regulated in response to glucose, participating to a translational reshaping to adapt beta cell metabolism. The early acute increase in the translation machinery components prepare the beta cell for further protein demand due to glucose-mediated metabolism changes.

## Introduction

Pancreatic islet β-cells play a pivotal role in the maintenance of normoglycemia by synthesizing, storing and secreting insulin. Glucose uptake and metabolism are essential for regulation of glycemia by stimulating insulin secretion and triggering specific gene expression. These processes have been widely studied in rodent since 1970s (1). Due to the long-standing difficulties to generate a human cellular model (2), or to access to primary human islet preparations derived from deceased donors (3), there is a scarcity of results obtained from human cells. In addition, despite many similarities, there are major differences between human and rodent models such as the copy number of *insulin* genes, the expression of different transcription factors in glucose-stimulated insulin secretion, the architecture of the Islet of Langerhans with functional implication and, finally, the susceptibility to β-cell injury (2). Moreover, concerning glucose-dependent gene expression regulation, recent transcriptome studies have demonstrated important differences in expression levels between human and rodent cell lines. In the rat β-cell line INS-1, more than 3700 genes were significantly affected in response to glucose (4). In contrast, a recent transcriptome study of the first human β-cell line (EndoC-BH1), able to secrete insulin in response to glucose stimulation (5), showed that only a scarce number of genes were modified at the mRNA level for cells exposed for eight hours either to high or low glucose concentrations (6). Accordingly, a previous report on donor human islet treated similarly during twenty-four hours (7) reported that the expression of only 20 genes was affected. Taken together, these findings highlight a considerable difference in gene expression regulation at transcriptional level between human and mouse pancreatic β-cell.

Glucose regulation has also been addressed at post-transcriptional level in rodent models since the 70s. In particular, attention was focused on the regulation of glucose-induced pro-insulin synthesis (8–10) reporting that the first cellular response to replenish insulin was entirely mediated at translational level without affecting mRNA abundance (8). Beside *pro-insulin*, synthesis of other proteins was also stimulated, but these proteins were not identified. A translatome study addressed this issue in mouse insulinoma 6 (MIN6) cell line by polysome profiling (11) and identified 313 mRNAs, for which the association with polysomes was changed by at least 1.5 times. Interestingly, in low glucose the Integrated Stress Response (ISR) mediates eIF2 phosphorylation, promoting translation of a group of mRNAs including b-zip transcription factors such as ATF4, CHOP (DDIT3), and c-Jun. The translation of these mRNAs was reduced upon glucose increase and eIF2 dephosphorylation, linking regulatory pathway activity and protein expression regulation (11).

To date, no translatome studies have been made on human β-cells to address glucose induced post-transcriptional regulation but a recent proteomic study showed that a four-hour glucose treatment of human pancreatic β-cells exhibiting glucose-inducible insulin secretion (12) induces an increase in proteins involved in energetics an insulin secretion (13). In the present work, these human pancreatic β-cells were exposed to high glucose concentrations for 30 min to observe the early cellular response. We observed a global protein synthesis increase, independent from transcription regulation. We identified 402 mRNAs that are differentially translated in response to glucose with different groups of co-regulated transcripts. We found mTOR and eIF2-sensitive elements in a majority of them and, accordingly, we found that both pathways activate translation of specific mRNAs in response to glucose. Upregulated genes are mainly coding for ribosomal proteins, increasing the translation machinery potential, allowing the cell machinery to be engaged in a metabolic response to glucose.

## Materials and methods

### Cell culture and treatment

EndoC- βH2 cells (12) were cultured in low-glucose (5.6 mmol/L) DMEM (Sigma-Aldrich) with 2% BSA fraction V (Roche-Diagnostics), 50 mmol/L 2-mercaptoethanol,10 mmol/L nicotinamide (Calbiochem), 5.5 mg/mL transferrin (Sigma Aldrich), 6.7 ng/mL selenite (Sigma-Aldrich), 100 units/mL penicillin, and 100 mg/mL streptomycin. Cells were seeded at a 40% confluence on plates coated with Matrigel (1%; Sigma-Aldrich), fibronectin (2 mg/mL; Sigma-Aldrich). Cells were cultured at 37°C and 5% CO_2_ in an incubator and passaged once a week when they were 90–95% confluent. For the polysome profile experiments cells were plated 4 days before treatment to reach 80-90% confluence the day of experiment. Cells were cultivated for 24 h at 0.5 mM glucose and were then treated with different concentrations of glucose to obtain media at 5.6 mM, high-glucose media at 20 mM or with low-glucose media at 0.5 mM for 30 min.

### Western blot & antibodies

Protein concentrations were quantified using a Pierce BCA Protein Assay Kit (Thermo Fisher Scientific). Proteins (20 μg) were resolved by SDS-PAGE and transferred to a membrane using an iBlot2 Gel Transfer Device (Thermo Fisher Scientific). Membranes were incubated with specific primary antibodies against: phospho-Ser52-eIF2α (SAB4300221 Sigma), eIF2α (SAB4500729 Sigma), tubulin (T9026 Sigma), phospho-4EBP1 (2855 CST), 4EBP1 (9644 CST (14),), Phospho-eEF2 (2331 CST), eEF2 (2332, CST), ATF4 (1815, CST), RPL7 (14583-1-AP, PTGlab). Membranes were incubated with species-specific horseradish peroxidase, or fluorescent–linked secondary antibodies (1:10,000) and visualized on a Odyssey Fc Dual-mode Imaging System Instrument (LI-COR). Quantification was done (**Figure S6**), and profiles of the **Figure 7** were obtained, using Image Studio Lite 5.2.5. These data were processed to generate graphical representation with statistics with Rstudio 1.2.1335.

### Polysome profiling

Polysome profiling was performed on three independent cell cultures both in high (20 mM glucose) or low (0.5 mM glucose) and each replicate corresponded to approximately 30 million cells. After the glucose treatment, cells were washed once in ice cold PBS containing 100 µg/ml Cycloheximide. The PBS was then removed, the lysis buffer (80 mM KCl, 10 mM Tris pH7.4, 5 mM MgCl2, 0.5% Triton X 100, 0.5% Na-Deoxycholate, 40U/µL RNAsin, 1 mM DTT) was added directly to the plate and cells were scraped and collected. After 10 minutes incubation on ice, the lysates were centrifuge at 10,000 x g for 5 min at 4°C. 10 A254 units of lysates were layered onto a 11 ml 20–50% (wt/vol) sucrose gradient prepared in the lysis buffer without Triton X-100. The samples were ultra-centrifuged at 39,000 × g for 2.5 h at 4°C in a SW41 rotor. The gradients were fractionated in 14 fractions of 0.9 ml using an ISCO fractionation system with concomitant measurement of A254. Polysome/monosome ratios were obtained by dividing the area of the polysomal peaks by the area of the peak for the 80S monosomes. Total lysates and fractions were supplemented with 50 µl of 3 M NH4Ac, 10 ng of Luciferase RNA (Promega), 1 µl of Glycoblue (Ambion) and 1.2 ml of ethanol. Samples were vortexed and precipitated overnight at −20°C. The pellets were collected by centrifugation at 10,000 × g for 10 min at 4°C, washed once in 75% ethanol and resuspended in 100 µl DEPC-treated H2O. Samples were then treated for 1 hour at 37°C with RQ1 DNase (Promega) to remove possible contamination by DNA. RNAs were isolated by acid phenol: chloroform and precipitated in 1 ml Ethanol supplemented with 50 µL 3M NaOAc pH 5.2, and 1 µl of glycoblue. Pellets were resuspended in 20 ul DEPC-treated H2O. For sequencing equal volumes of fractions were pooled: fractions 5-6 (Monosomes), fractions 7-9 (Light polysomes) and fractions 10-13 (Heavy polysomes). Quality and quantity of pooled fraction was tested by the bioanalyzer RNA 6000 Pico kit (Agilent). Sequencing was performed by the Genom’ic platform (Institut Cochin, Paris). Libraries were prepared using TruSeq RNA Library Preparation Kit (Illumina) with rRNA depletion using Ribo-zero rRNA removal kit (Illumina) following manufacturer’s instruction. High-throughput sequencing was performed using Hiseq 2000 (Illumina) system for 75nt single-end reads.

RNAs were extracted using RNeasy mini kit (Qiagen, ref: 74104), DNAse treatment was performed with RNAse-free DNAse Set (Qiagen, ref: 79254). Equal volumes of all samples were reverse transcribed with Superscript IV reverse transcriptase (Life Technologies) for polysomes samples (**Figures S2C, D**). Reverse transcripts were obtained using RNA at 1ug/50ul with the kit High Capacity cDNA RT (Life Technologie ref: 4368814) for total RNA samples (**Figure S1C**). qPCR was done with GoTaq qPCR Master Mix (Promega ref: A602) on ViiA 7 Real-Time PCR System (Thermo Fisher Scientific). DNA contamination was assessed omitting the RT, no significant signal was obtained. Custom primers were designed with the tool developed by Integrated DNA technologies (IDT, https://eu.idtdna.com/scitools/Applications/RealTimePCR/), and their efficiency was determined following serial dilutions of cDNA samples. Primer sequences: PTPRN, Fw (5’-3’): GTCTCCGGCTGCTCCTCT, Rv (5’-3’): GCCTGCGGTCAAATAGACA; CHGA, Fw (5’-3’); CAAACCGCAGACCAGAGG, Rv (5’-3’); TCCAGCTCTGCTTCAATGG; Cyclophilin-A primer sequences used for normalization, Fw (5’-3’): ATGGCAAATGCTGGACCCAACA, Rv (5’-3’): ACATGCTTGCCATCCAACCACT; CCNG1, Fw (5’-3’):GATATCGTGGGGTGGGTGA, Rv (5’-3’):TCAGTTGTTGTCAGTACCTCTATCATC; Hist1H3C, Fw (5’-3’): GCTTGCTACTAAAGCAGCCC Rv (5’-3’): AGCGCACAGATTGGTGTCTTC; Hist1H3D, Fw (5’-3’): CCATTCCAGCGTCTAGTCCG, Rv (5’-3’): TCTGAAAACGCAGATCAGTCTTGPTPRN.

### Bioinformatic analysis of transcriptome and polysome sequencing

Sequencing libraries were prepared from three biological replicates for both conditions (0.5 mM and 20 mM glucose). We prepared triplicates for high and low glucose conditions to produce RNA-seq libraries containing between 16-18 million reads for transcriptome and 8-14 million reads for polysomes pools. Almost 60% of reads on average were uniquely mapped to the human genome (**Figures S1D**, **S2A**). Reads mapped to genes annotated as “protein coding genes” were kept for further analysis. Lowly expressed genes, frequently associated with high variability between replicates, were discarded. Samples from different conditions were grouped together by hierarchical clustering and PCA (**Figures S1E**, **S2B**), ensuring reproducibility of our replicates. As described below, we thereafter proceeded with differential gene expression analysis using the limma R package (15).

#### Pre-processing

Raw fastq file obtained from the sequencer were firstly checked for their quality using FastQC and reporting with MultiQC v1.6 (16). No reads were discarded nor trimmed.

#### Mapping, read counts and TPM calculation

Quality controlled reads were then mapped to the human genome (GRCh38 from Ensembl 92) using STAR 2.6 (17) by using the parameter –quantMode GeneCounts to generate gene counts tables. STAR aligner was further instructed to generate an output (–quantMode TranscriptomeSAM) suitable as an input for RSEM (18). RSEM reports tables with transcript per million (TPM) for genes and mRNA isoforms (19). For all the rest of downstream analyses, the tables were filtered to retain only the genes which are annotated as protein coding in the Ensembl 93 annotation tables.

#### Filtering of lowly expressed genes

Gene counts table were transformed in log CPM (Counts per Million base). Genes whose CPM values were smaller than 1 at least in one sample were discarded. Then a customized R function further filtered genes whose coefficient of variation (defined as the ratio of the standard deviation over the mean) within replicates was lower than 0.75 and the mean CPM expression was higher than 4. As a first step for quality control of our datasets we performed a hierarchical clustering analysis by using the TPM tables for each sample. Clustering was performed on the Euclidean distance matrix and the Ward’s minimum variance method was used for forming clusters (option Ward.D2 in the hclust function of R).

#### Differential expression analysis and clustering

Analyses were performed using the limma (15) Bioconductor package. Differentially expressed genes (DEGs) or translated genes (DTGs) were identified by fitting linear models between all the pairs of the three polysome profile fractions applying the ebayes method to calculate p-values. Only genes with adjusted p-values for multiple testing ≤ 0.05 were selected. A separate list containing the TPM (transcripts per million reads) values was kept for downstream analysis. The average expression of each gene was calculated in each fraction (monosomes, light polysome and heavy polysomes) and condition (low or high glucose). Then for each fraction and gene the logarithmic ratio of means of high glucose over low glucose was calculated (log ratio of mean TPM expression). The generated log ratio matrix was then used in our integrative clustering approach which comprised the application of 3 clustering algorithms. Hierarchical clustering (hclust), k-means clustering (kmeans) and a model based bayesian approach clustering (mclust) were applied to the log ratio matrix of translation. Based on the silhouette measure for each clustering we evaluated that the mclust method represents better the structure of the translation data set.

#### mRNA features collection

We developed an in-house software for the retrieval and calculation of an array of sequence features for a given set of genes, The RNA features extraction tool is freely accessible in the GitHub repository (https://github.com/parisepigenetics/rna_feat_ext). The tool searches for either the most well annotated transcript for each gene in the gene list or it can also choose the most expressed transcript if a transcript abundance file is provided (e.g. the output of tools like RSEM or StringTie). We used the latter possibility using the average of all the polysomal samples. For each transcript, the tool extracts different mRNA features using the bioMart API. The mRNA features that we extracted were: length of the 5’UTR, CDS and 3’UTR and the GC content of both 5’ and 3’ UTR. The software also calculates the folding free energy for the 5’ and 3’ UTR (by using the RNAfold algorithm of the Vienna package (20), normalized by the length (MFE per bp) that is a measure of the stability and the complexity of the RNA secondary structure, an “in-house” devised TOP-mRNA local score and the Codon Adaptation Index (CAI (21),), based on the codon usage of human genes. All statistical analyses of the features distributions along different translation behaviours were conducted by the groups based statistics R package *ggstatsplot* (https://cran.r-project.org/web/packages/ggstatsplot/index.html) using the Kruskal-Wallis H-test for comparing independent samples.

#### Enrichment analyses

We perform an array of different enrichment analyses as they are included in the clusterProfiler and DOSE R packages (22, 23) including Gene Ontology annotation analysis (for all “biological processes”, “molecular functions” and “cellular component” categories), gene set enrichment analysis GSEA, KEGG pathway analysis (24) and Reactome pathway analysis (25). We visualise the results of the most significant enrichment on categories, gene sets and pathways by using typical bar/dot-plots of enrichment and a powerful graphical output of the above R package, the Gene-concept network plot (cneplot from clusterProfiler).

#### Translation ratio, translation efficiency

We define and calculate for translation ratio by using the measure of stable state mRNA from RNA-seq and a measure for mono/polysome fraction occupancy from polysome profile. We first computed the average of all the 6 polysome profile conditions (mono-, light- heavy- in high and low glucose) and the average of the two RNA-seq conditions (high and low glucose) and then we simply divide each polysome profile condition average with the respective RNA-seq average. This calculation resulted in 6 measurements of translation ratio for all genes in the three polysome fractions (monosomes, light and heavy polysomes) and the two glucose treatments (high and low). Then we computed what we call the translation efficiency of each gene in both glucose treatments (high and low) by subtracting the average of translation ratio in light plus heavy polysomes in high glucose from the same average in low glucose. These calculations allowed us to distinguish the most translated genes and those ones with the biggest shift in translation between high and low glucose. We have classified differences in translation ratios between high and low glucose to 3 groups according to the log fold-change (FC). First at +0.5 LogFC as highly translated in glucose, second with -0.25 logFC as lowly translated and a control group with +/-0.01 LogFC as control. We choose these thresholds in such a way as to generate 3 groups almost equal (UP, DOWN and control) sizes so that the comparative statistics will be more robust.

## Results

### Glucose induces an acute increase in protein synthesis in human β-cells

**Figure 1:**
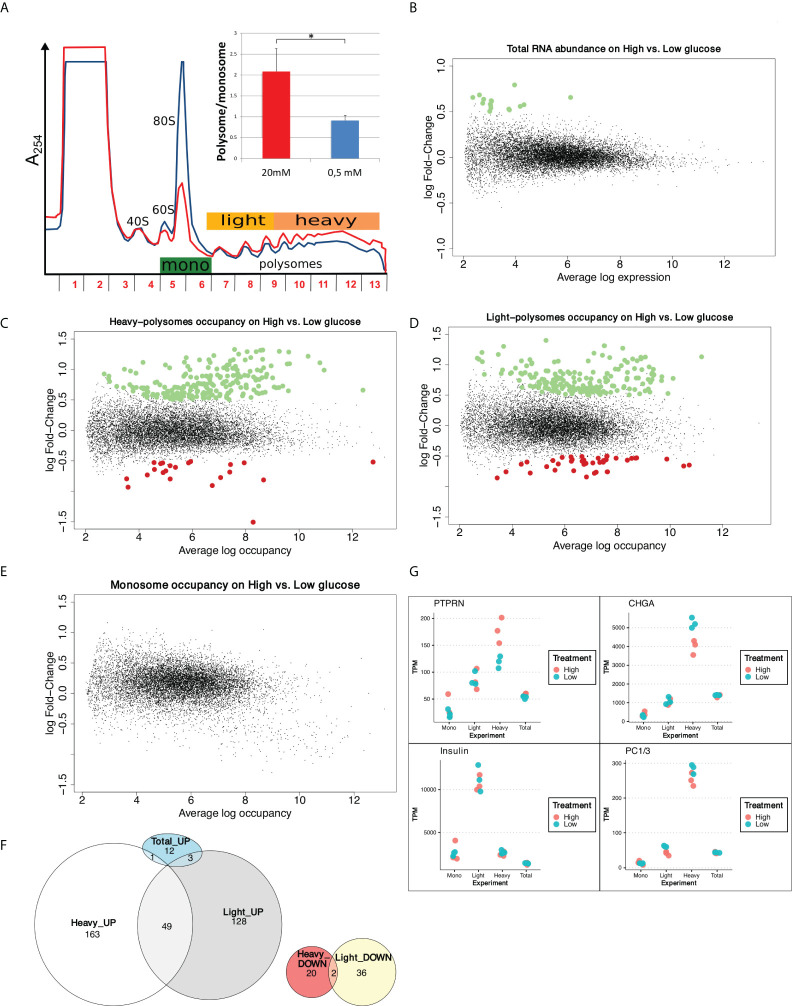
Glucose induces increase in protein synthesis without affecting mRNA abundance and regulates translation rates of a subset of mRNAs. **(A)** Polysome profiles of EndoC-βH2 with Low glucose (blue) and High glucose (red). The absorbance at 254 nm (A254) recorded during the collection of the fractions of the gradient is displayed. The positions of 40S, 60S, 80S and polysomes are indicated. The tree-colored bars represent the fractions that were pooled for sequencing: monosomes (green), light polysomes (yellow), heavy polysomes (light orange). EndoC-βH2 cells in high glucose had a significantly higher polysome/monosome ratio than did EndoC-βH2 cells in low glucose. Cells were treated in parallel using paired culture plates, and centrifugated together in the same rotor. Figure shows a representative replicate. The statistical significance of the polysome/monosome ratio was assessed using a paired t-test from three independent experiments (*p < 0.001, n = 3). **(B)** MA-plot of total mRNA abundance, dots in green specify up-regulation. **(C–E)**. MA-plots for each pool of fractions: heavy polysomes **(C)**, light polysomes **(D)** and monosomes **(E)**; dots in green corresponds to transcripts that are upregulated upon glucose stimulation, dots in red to transcripts that are downregulated. **(F)** Venn diagram showing the overlap between glucose UP- or DOWN- regulated transcripts in Light and Heavy polysomes. Total_UP are transcripts varying in abundance. **(G)** TPM values for each condition and each replicate are plotted. In red High glucose replicates, while in cyan Low glucose replicates. Names of the plotted gene is indicated above.

To monitor and quantify the translation activity in response to glucose, we performed a polysome profiling analysis on a functional human β-cell line previously fully characterized as a valid model of human beta cells, that is able to produce insulin in response to glucose (EndoC-βH2 cells, see Material and Methods, and (26)). Polysome profiling is a gold standard technique to analyze cellular translation activity. Ribosome complexes of different densities are separated on a sucrose gradient consistent with the number of ribosomes. Briefly, EndoC-βH2 cells were cultured for 24 hours in 0.5 mM glucose and then treated for 30 min with either 0.5 mM or 20 mM glucose. Polysome profiling showed that increasing glucose to 20 mM triggered an important increase in the content of polysomes in parallel to a decrease of the monosomes peak (80S) (**Figure 1A**). Polysome/monosome ratio was raised 2 times, which is classically observed with a global increase in mRNA translation. This translation upregulation was confirmed by ^35^S-Met incorporation (**Figure S1A**).

We next quantified whether the global increase in translation was associated to a modification in mRNA steady state levels by genome-wide transcriptome analysis. As illustrated by the MA plot in **Figure 1B**, global mRNA levels were not significantly affected by a 30 min glucose treatment. Only 16 transcripts (**Figure S1B**) were found to be significantly affected, 7 of them coding for histone proteins. However, these variations are low, with a fold change of less than 2-fold. We validated this result by RT-qPCR showing that *Histone1H3C* and *Histone1H3D* mRNAs increased from 30 min after glucose shift (**Figure S1C**), in an extent similar to that observed by RNAseq (log FC = 0.7 at 30 min, **Figure S1B**, corresponding to FC = 1.6). Conversely, the abundance of mRNAs such as, *PTPRN*, *CHGA* or *CCNG1* was not significantly affected even after 1h or 2h after the glucose shift. This finding is in agreement with the results described in Richards et al., showing that even 8 hours after glucose increase, only a scarce number of genes were modified at the mRNA level (6). Hence, the global increase in protein synthesis is virtually independent from transcription regulation.

In conclusion, we show here for the first time that human beta cells respond to glucose by a rapid and important increase in mRNA translation, which is virtually independent of changes in the transcriptome.

### Glucose regulates translation rates of mRNAs involved in the insulin secretion pathway and in the translation machinery

We next studied genes whose translation rates are regulated in response to glucose by sequencing the mRNAs associated with monosomes and polysomes (**Figure 1A**, see Materials and Methods). The commonly adopted strategy to identify translated genes is usually to consider mRNAs associated with more than 3 ribosomes (27), but each fraction from monosomes to heavy polysomes could also be sequenced (28). To obtain a good compromise between resolution and sensitivity, we analyzed separately the monosomes (80S), light polysomes (2-4 ribosomes per mRNA), and heavy polysomes (> 4 ribosomes per mRNA). Accordingly, fractions were pooled (see **Figure 1A**) to collect monosomes (fractions 5-6), light polysomes (fractions 7-9) and heavy polysomes (fractions 10-13) before RNA sequencing.

Differential analysis using the limma package (15) highlighted that the abundance of 235 mRNAs in heavy polysomes and 218 in light polysomes was significantly changed upon glucose shift (adjusted p-value < 0.05 and logFC > 0.5, **Figures 1C, D**), but not in monosomes (**Figure 1E**). These changes do not correlate with a significant difference in mRNA total level (**Figure 1B**). As expected from the global increase in translation we observed in high glucose (**Figure 1A**), most of the identified transcripts were enriched in polysomes (**Figure 1F**): 164 in the heavy polysomes, 131 in the light polysomes, 49 common to both. Thus, only a minority of transcripts, 58 in total, were downregulated. Interestingly, from the 16 transcripts increased in RNA abundance (**Figure 1B**) only four have been found increased in polysomal fractions (**Figure 1F** and **Figure S1B**). Except for these four genes, the variation for each transcript observed in polysomal fractions corresponds to a specific translational regulation independently of any variation in mRNA abundance and, consequently, of any transcriptional regulation.

Continuing the investigation of differentially translated genes, we proceed by assessing if glucose stimulation modified the biosynthesis of insulin and of known major factors involved in insulin maturation and secretion pathway. We focused on gene products whose biosynthesis was reported to be enhanced in response to glucose in rodent cells, such as Protein Tyrosine Phosphatase Receptor Type N (PTPRN) (29), chromogranin A (CHGA) (30), and pro-hormone convertases 1/3 (PC1/3) (31). In heavy polysomes, *PTPRN* mRNA was enriched while *CHGA* mRNA was reduced (**Figure 1G** and **Figures S2C, D**). Interestingly, the relatively short *Insulin* transcript associates mainly with light polysomes (**Figure 1G**), as observed previously in a mouse cellular model (11).

Our data show that the human EndoC-βH2 beta cell line incubated with high glucose for 30 min quickly modify the translation rates of at least two mRNAs that code for proteins involved in the insulin secretion pathway. In contrast, we did not find any particular expression difference in the dot plot for *Insulin* and *PC1/3* mRNAs (**Figure 1G**), and accordingly we did not find these genes as differentially translated in our conditions.

**Figure 2:**
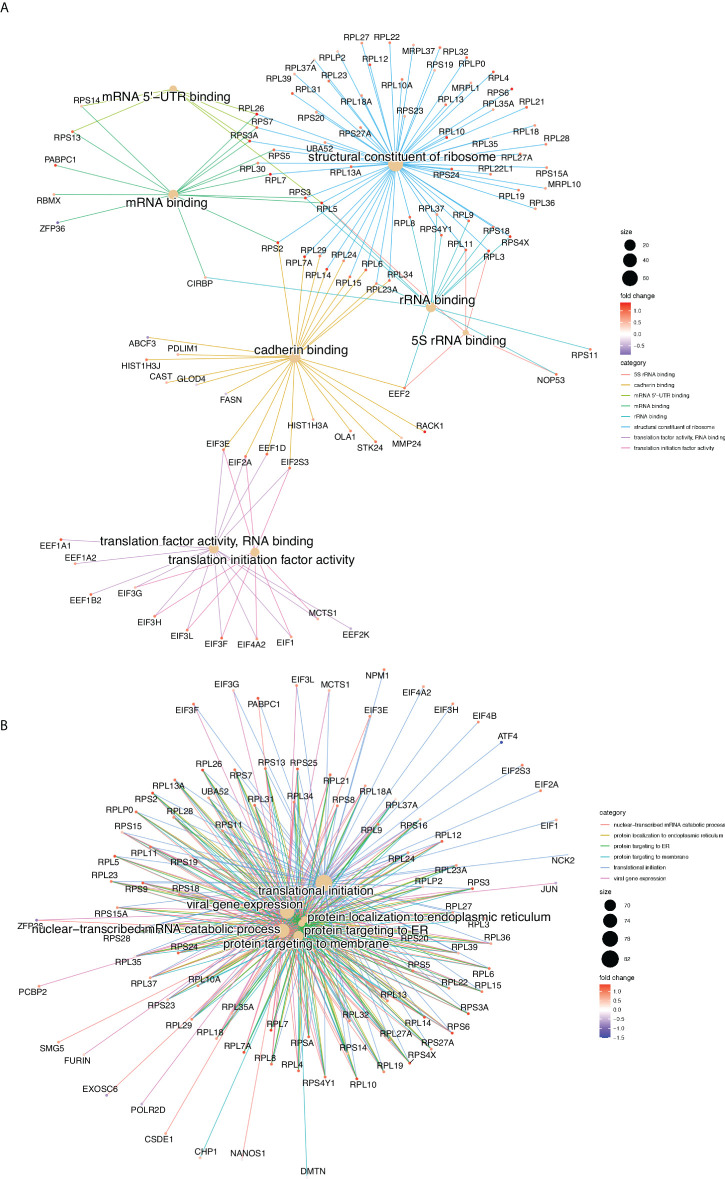
Gene Ontology enrichment analysis, gene-concept plots (cneplots). Each cneplot illustrates the most significantly enriched Molecular Functions (MF, **A**) and Biological Processes (BP, **B**) categories. Lines of the same colour connect genes of the same GO category and the colour gradient of each gene corresponds to its log-FC.

Gene Ontology (GO), gene set and REACTOME pathway enrichment analyses were performed for all the Differentially Translated Genes (DTGs). **Figure 2** illustrate the gene-concept plots (cnetplots) of each ontology term and all its associated genes of the top 10 enriched categories for molecular functions (MF, **Figure 2A**) and biological processes (BP, **Figure 2B**). Cnetplots are an informative way to represent the relationships between different ontology terms in a graph of all the associated genes together with the logFC for each gene. It is evident that the majority of the translationally upregulated genes (red nodes) are enriched in GO terms related to the biosynthesis and metabolism of proteins, rRNAs and to the translation machinery (**Figure 2**). **Figure S2E** illustrates the top 20 REACTOME pathways that are enriched in DTGs which further corroborate the finding of the GO analysis (**Figure 2**).

In conclusion, our results show that glucose concentration changes modulate translation rate of specific mRNAs of the insulin secretion pathway and promotes the synthesis of the translation machinery components.

### Differentially translated mRNAs in response to glucose share unique structural and sequence features

**Figure 3:**
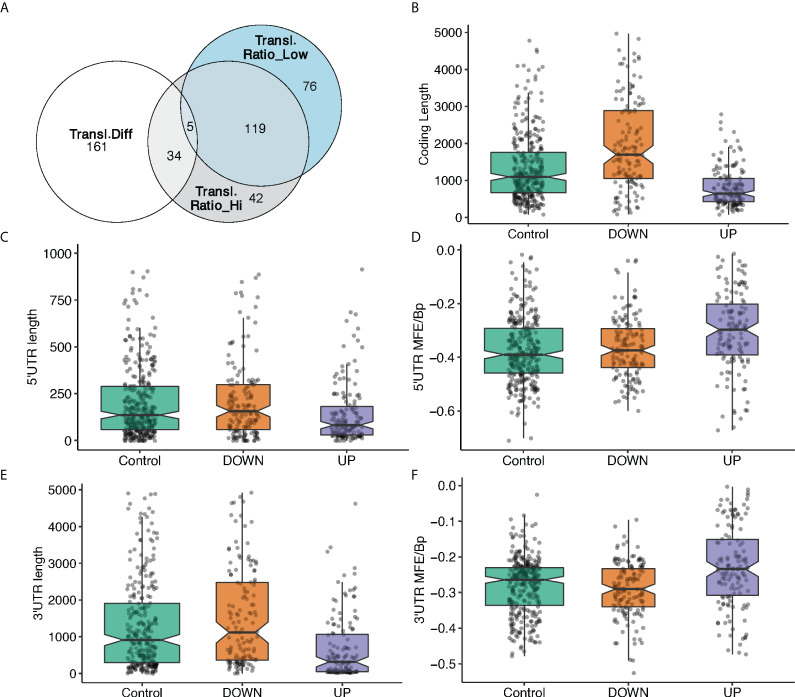
mRNA feature analysis of the 3 groups of differentially translated mRNAs. **(A).** Venn diagram showing the overlap between the 200 mostly translated genes: best translated in high glucose (grey circle), best translated in low glucose (blue circle), mRNAs with the most difference ones in ranking between low and high glucose (white circle). **(B-F).** mRNA features analyses of 3 groups of mRNAs based on the difference in translation ratio between high and low glucose. Higher (UP) or the lower (DOWN) translation ratio in high glucose, and a control group with no significant changes between high and low glucose. MFE/Bp is the folding free energy normalized by the length (see Material and Methods). **(B),** coding length; **(C),** 5’UTR length, **(D),** 5’UTR MFE/Bp, **(E),** 3’UTR length, **(F),** 3’ UTR MFE/Bp.

It might be tempting to explain the translation modulation of specific mRNAs in response to glucose by a simple global translation increase. To test this, we calculated the translation ratio, defined as the abundance ratio of the translating mRNA in mono/polysome fractions to total mRNA regarding a certain gene (see Material and Methods for detail), for the most translated transcripts for each gene (**Table S1**), and then asked if the 200 most translated mRNAs are the same in low and high glucose (**Figure 3A**), by ranking them according to the translation ratio. We found that around 40% (76) of the best translated transcripts in high glucose (**Figure 3A**, grey circle) were not among the best translated in low glucose (**Figure 3A**, blue circle). Furthermore, 80% of the mRNAs that display the major differences in ranking (**Figure 3A**, white circle) are not found in the 200 top translated mRNAs in high or low glucose. The cellular response to glucose is therefore not just a simple increase in overall translation.

Translational adaptation to glucose seems therefore to be a complex process. Thus, we further investigated potential association between the differential translation regulation and specific sequence and structural features of the DTGs. To this end, we have classified differences in translation ratios between high and low glucose to 3 mRNA groups: 147 mRNAs with higher translation ratio in high glucose, 137 with a lower translation ratio in high glucose and a control category of 320 mRNAs with no significant change between high and low glucose. The most translated transcript of each of these 604 genes was analysed by our in-house developed software (detailed in Materials and Methods) to retrieve sequence information from ENSEMBL database and identify structural features. Several characteristics appear in the statistical analysis of the features (**Figures 3B-F**, **Figure S3**). Among them, the minimum folding energy normalized over the length of the sequence (MFE per BP) is an interesting measure to estimate the complexity of an mRNA untranslated region (UTR) structure.

Transcripts with higher translation ratio difference between high and low glucose appear to have statistically significant shorter open reading frames (ORFs), and shorter and less complex UTRs. Conversely, transcripts that are down regulated have longer ORFs and more structured 3’UTR since their MFE per BP distribution slightly decreases (**Figures 3B-F**).

These results indicate that the sequence and structural features we have used allow the classification and characterization of the highly translated mRNAs; so they provide a good tool to dissect the different classes of translational behaviour of transcripts in mono-, light- and heavy- polysomes. Motivated by that, we proceeded with the dissection of this behaviour and the sequence-structure characterization of the different transcript classes.

### Differentially translated mRNA clusters display specific mRNA features

**Figure 4:**
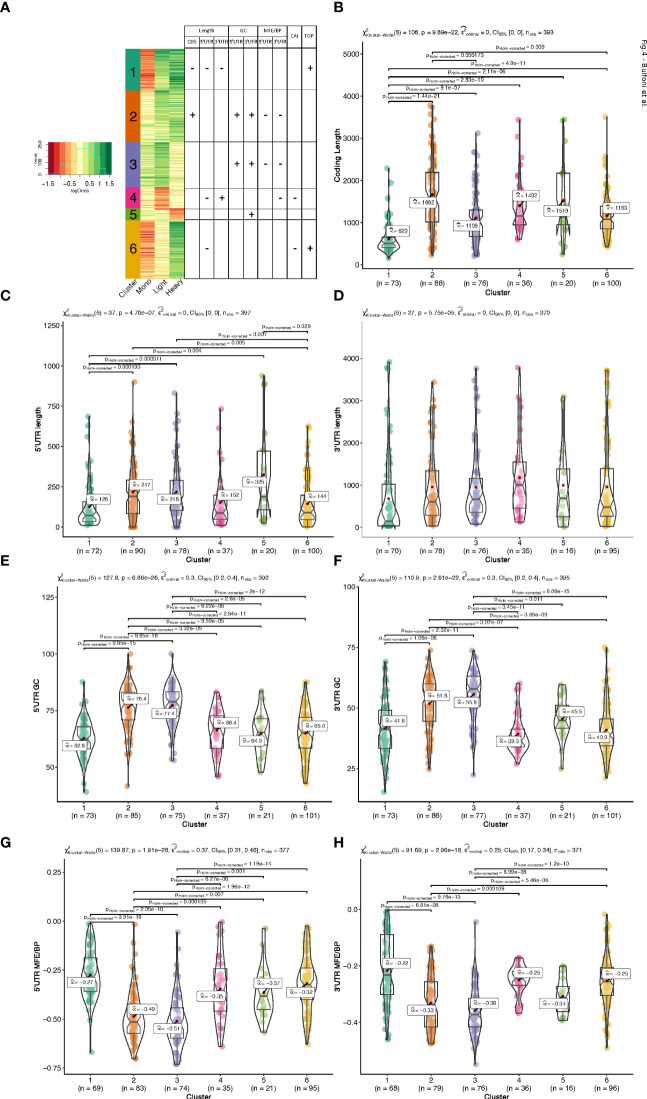
Clustering and mRNA features analysis. **(A).** Heatmap of the log2 ratios between the average TPM of each differentially translated gene in high- over low- glucose. An unsupervised model clustering algorithm was used to cluster the differentially translated genes into six groups represented here with the coloured vertical bar at the left side of the figure. Colours range from dark red when genes are less represented in a polysome fraction to dark green when overrepresented in a polysome fraction in high glucose condition. The table summarizes the variations observed for the different features presented in **(B-H)** each box plot corresponds to an analysis of a particular feature for all the 6 clusters of translation behaviour. The Kruskal-Wallis H-test is used for all the group comparisons to test for median differences with the Dunn test for each pairwise comparison. The test statistics are shown in the subtitle and each significant pairwise comparison (corrected for multiple testing) is plotted only for significant pairwise differences.

To group the translationally co-regulated transcripts, we clustered the 402 differentially translated mRNAs we identified previously (**Figure 1F**) based on their behaviour between monosomes, light, and heavy polysomes. To this end, we calculated for each mRNA the log2 ratio of the average abundance in high over low glucose condition for each of the three ribosomal fractions. The log ratios matrix was then subjected to a modelling clustering method able to determine in an unsupervised way the best model that characterizes the data (32). The model generated six clusters, which highlighted six different types of behaviours (**Figure 4A**, coloured bars with the cluster number). Genes of the top cluster (n°1, seagreen bar) showed a clear pattern for 73 mRNAs shifting from the monosomes to polysomes (both light and heavy), which is the expected behaviour for a mRNA with increased translation. Cluster n°6 (102 mRNAs) presented a behaviour similar to cluster 1 with an increase of mRNAs in heavy polysomes but without any rise in the light polysomes. These transcripts move from the monosomes to the heavy polysomes fraction and correspond to transcripts whose translation is greatly increased. Instead, the mRNAs of clusters 2 and 3 (90 and 79 mRNAs, respectively) showed increased mRNA levels for light/heavy and monosome/light polysomes, respectively. We reasoned that at low glucose concentration these mRNAs are associated with small complexes that have a density smaller than one ribosome and correspond to mRNAs newly recruited to the translation machinery. Finally, clusters 4 and 5 (37 and 21 mRNAs, respectively) collected all the mRNAs whose levels in the polysome fractions decreased upon glucose stimulation, which might reflect a decrease in their translation. The difference between these two clusters comes from the fact that the decrease is observed in light polysomes for cluster 4 and in heavy polysomes for cluster 5.

**Figure 5:**
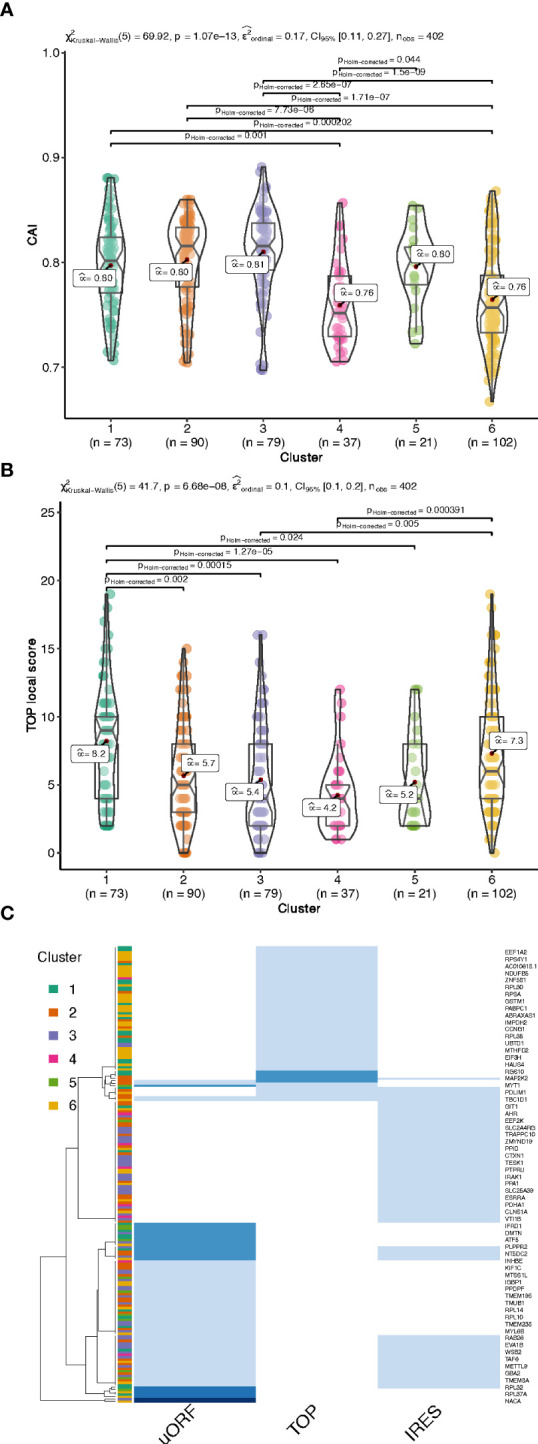
Codon Adaptation Index (CAI, **A**) and functional motifs analysis of the UTRs by interrogating the UTRdb for TOP motifs **(B)**, and TOP, uORF and IRES in the 5’UTRs **(C)**, common gene names of the encoding gene of each transcript are indicated on the right side. The darker the blue, the higher the number of features per RNA (from 1 to 3 per RNA).

Based on the link between features and behaviours that we observed (**Figure 3**), we hypothesized that the different patterns observed for the clusters could be a consequence of *cis*-regulatory elements present on the mRNAs which could serve as binding sites for *trans-*acting factors such as RNABPs and miRNAs. To investigate this possibility, we calculated a series of mRNA features for the most abundant transcript for each gene. We developed an RNA feature extraction tool (see Material and Methods) that is able to download, from the ENSEMBL database bioMart (33), the transcript sequences with additional annotations and calculate sequence and structural properties (**Table S2** for the full table of results). We proceeded by grouping these mRNA features between the 6 clusters of different translation behaviour identified by the model clustering approach (**Figures 4**, **5**, and **Figure S4**). Strikingly, the length of the coding sequence (CDS) of cluster 1 mRNAs was significantly shorter, while in cluster 2 the CDS were longer (**Figure 4B**). Next, we analysed the length of the UTRs (**Figures 4C, D**). Notably, there was a tendency for mRNAs of cluster 1 to have shorter 5’ and 3’ UTRs than the other clusters. Cluster 4 and 6 showed similar tendencies towards shorter 5’ UTRs (**Figure 4C**). Cluster 4, that corresponds to mRNAs whose translation decreases in response to glucose, contained longer 3’ UTRs (**Figure 4D**).

We next analysed the GC-richness of the 5’ and 3’UTRs, as a proxy to determine the complexity of secondary structures formed by the mRNA UTRs. Cluster 2 and 3 contained mRNAs with higher GC content on both 5’ and 3’ UTRs than the others (**Figures 4E, F**). In accordance, clusters 2 and 3 contained mRNAs with more structured 5’ and 3’ UTRs than the other clusters (**Figures 4G, H**). In addition, cluster 5, the cluster with the underrepresented mRNAs in the heavy polysome fraction, also appears to have the next higher GC content and lowest MFE per BP on its 3’UTR (**Figure 4F, H**), indicating that the 3’UTR structural and sequence complexity affects the translation regulation.

In addition to the UTR structure, we found a tendency towards a lower Codon Adaptation Index (CAI) for cluster 4 and cluster 6 (**Figure 5A**). It is interesting to note that a lower CAI is associated with slower translation rates and could participate to the accumulation of transcripts in monosomes (cluster 6) or light polysomes (cluster 4) rather than in heavy polysomes in low glucose (**Figure 4A**), even if this hypothesis would require further specific experiment.

We then searched for functional motifs in the UTRs by interrogating the UTRdb (http://utrdb.ba.itb.cnr.it/, 34), and we found already characterized RNA binding motifs in the UTRs of the mRNAs which are differentially translated (**Figure S5**). Interestingly, we found TOP motifs (5’-terminal oligopyrimidine (TOP) (35)) mostly represented in cluster 1 and 6 (**Figure 5B**), and also uORFs, and IRES features (**Figure 5C**). It is interesting to note that uORF and TOP features are mutually exclusive (**Figure 5C**), meaning that these features could be used independently to differentially regulate mRNA translation.

Taken together, the results suggest that the translational regulation of differentially translated mRNAs upon glucose concentration shift is strongly associated with, and can be characterized by, specific sequences as well as structural features on the UTRs and coding regions of these mRNAs.

### Clustering reveals that specific pathway regulators are co-regulated in response to glucose, and that TOP motifs are preponderant to upregulate the translation machinery components

**Figure 6:**
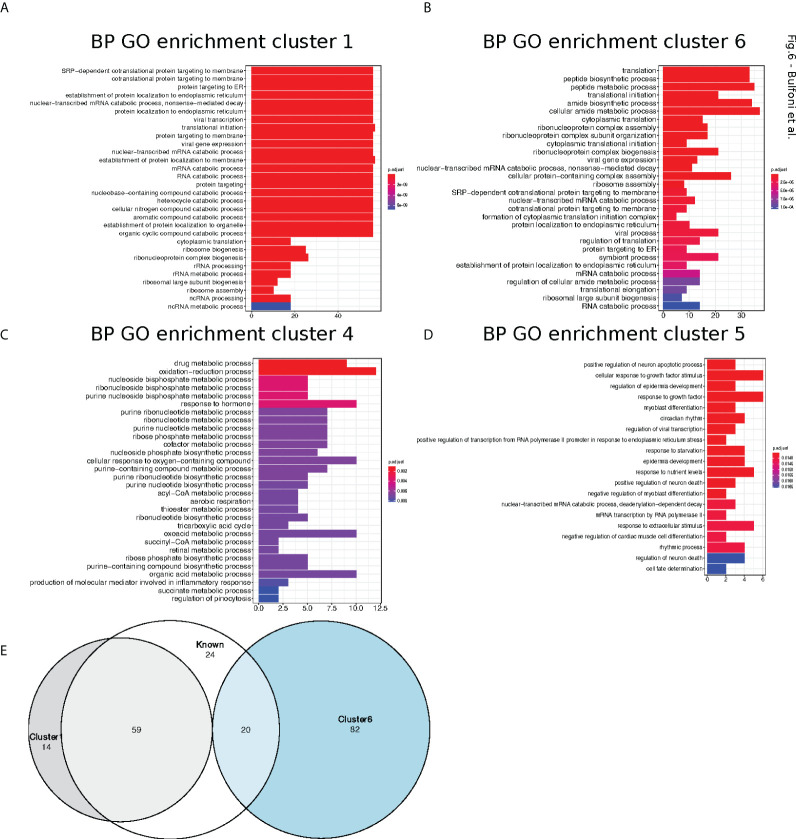
Cluster Gene Ontology enrichment. **(A–D).** barplot of gene ontology terms enriched for biological processes for clusters 1, 4, 5 and 6 using the R package clusterProfiler. **(E).** Venn diagram of genes belong to cluster 1 (grey circle) and 6 (blue circle) with previously reported TOP-RNAs (white circle).

**Figure 7:**
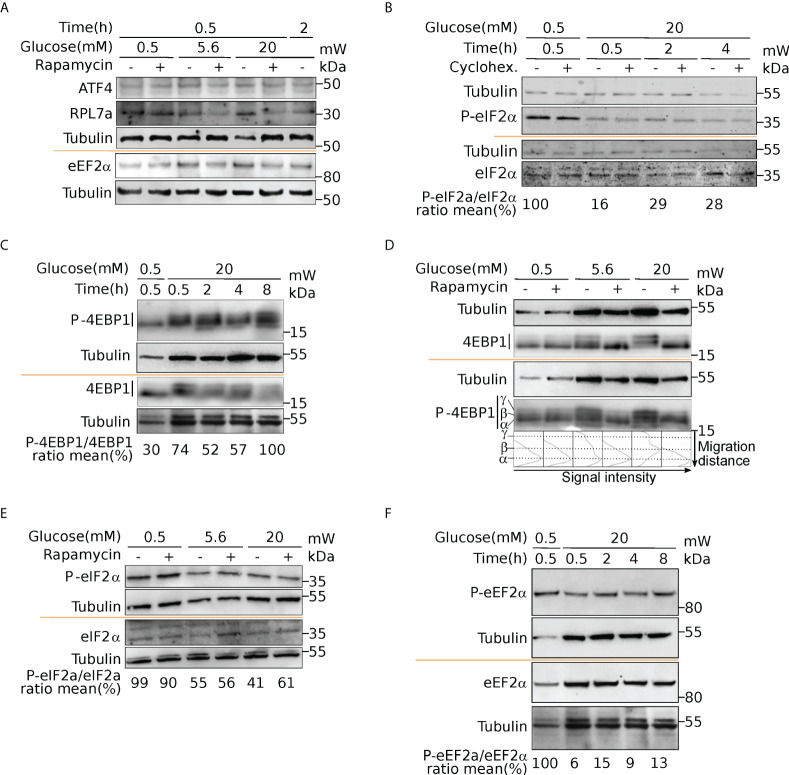
Activation of mTOR and eIF2α upon glucose induction. Western blot analysis of ATF4 and RPL7a (A), eEF2α and P-eEF2α (A, F), eIF2α and P-eIF2α (B, E), 4EBP1 and P-4EBP1 (C, D). Cells were incubated with 0.5- or 20-mM glucose for the indicated time. Where indicated, cells were pre-treated for one hour with cycloheximide (CHX) to block translation, or with rapamycin to inhibit mTOR activation (see text). For 4EBP1, upper bands (b and g) correspond to hyperphosphorylated forms. The ratio phosphorylated/total proteins was reported from quantification presented in **Figure S6**.

Next, we performed gene ontology enrichment analyses, using the R package ClusterProfiler (22), in order to assess if the mRNAs belonging to the different clusters were implicated in specific biological processes. Cluster 1 and 6 were enriched for categories related to translation but especially for ER-related translation (**Figures 6A, B**). Cluster 4 (**Figure 6C**), which contained the translationally repressed mRNAs in high glucose, was enriched in mRNAs coding for proteins involved in regulation of metabolic processes and cell death. In particular, genes involved in tricarboxylic acid, acyl-CoA, and thioester metabolisms were found to be enriched in cluster 4. Cluster 5 with also repressed transcripts in high glucose was enriched for categories related to response to starvation and growth factors (**Figure 6D**), including mRNAs coding for b-zip transcription factors such as ATF4, DDIT3(CHOP), and c-Jun (**Table S2**) that had a similar behaviour in mouse beta cell (see introduction (11). Accordingly, we observed by Western blotting that ATF4 protein tends to decrease after a two-hour glucose shift (**Figure 7A** and **Figure S6**).

We thus investigated if ribosomal proteins or translation factors were found in clusters 1 and 6. We found that cluster 1 contained 56 mRNAs coding for ribosomal proteins (**Table S3**, RPL and RPS), corresponding to 70% of the total number of RP transcripts, while cluster 6 contained 13 mRNAs coding for eukaryotic translation factors of which 4 were elongation factors (eEF1A1, eEF1A2, eEF1B2, eEF2) and the remaining were initiation factors (eIF2A, eIF2S3, eIF3E, eIF3F, eIF3G, eIF3H, eIF3L, eIF4A2, eIF4B) (**Table S3**, eIFs and eEFs). As expected, we observed by Western blot that the protein amount of RPL7 and eEF2 are increased after glucose shift, and that rapamycin prevents this effect (**Figure 7A** and **Figure S6**). These mRNAs are known to contain the TOP motif (TOP-RNAs (35–37), that we found enriched in the differentially regulated mRNAs (**Figure 5B**), mostly in cluster 1 and at a lesser extent in cluster 6. Therefore, we compared the mRNAs found in cluster 1 and 6 with the list of mRNAs that were previously reported or proposed to be TOP-RNAs in three already published studies (35–37) (**Table S4**). Overlap between the three set of genes showed that cluster 1 contained 59 (58%) of the known TOP-RNAs while cluster 6 contained 20 (20%, **Figure 6D**). 80% of the mRNAs of cluster 1 are known to be TOP-RNAs, one hypothesis is that the similar behaviour of the cluster1 mRNAs is due to their TOP motif. We calculated the TOP local score for the 14 remaining mRNAs and found a similar distribution to cluster 1.

In conclusion, we found that translation increased for more than 70% of the mRNAs coding for ribosomal proteins, and also for genes involved in specific metabolic processes, response to growth factors or that regulate cell death.

### mTOR and eIF2alpha pathways are regulated upon glucose induction

We next sought to identify the molecular pathways driving such translation regulation in response to glucose in human beta cells. Our data unraveled a group of uORF-containing mRNAs that are translationally regulated upon glucose induction. uORF-mediated translation mechanisms involve the phosphorylation of eIF2 complex alpha subunit (eIF2α) on Ser51, which inhibits the assembly and recycling of the translational ternary complex (eIF2-GTP·Met-tRNAi), and results in a reduction in translation initiation. We therefore examined the phosphorylation status of eIF2α. Western blot analyses highlighted a dephosphorylation of eIF2α in response to glucose (**Figure 7B**, P-eIF2α, and **Figure S6D** for western blot quantification), indicating an increased availability of the ternary complex. Furthermore, adding the translation inhibitor cycloheximide does not prevent the activation of eIF2 through dephosphorylation, which is therefore independent of protein neosynthesis.

It has been described that glucose deprivation is sensed by aldolases, which cause the formation of a membrane-associated lysosomal complex, which in turn activates AMP-activated protein kinase (AMPK) (38). AMPK induces inhibition of mammalian TOR complex 1 (mTORC1) activity by phosphorylation of the tuberous sclerosis protein 2 (TSC2) tumor suppressor and the mTOR binding partner Raptor (39, 40). Since mTORC1 is a well-known regulator of protein synthesis, we postulated that upon glucose increase, mTORC1 activation participates in translation upregulation. It is well established that mTORC1 regulates translation through the modulation of the phosphorylation status of 4EBP1 (41). Thus, to further validate that mTORC1 signaling is a key player in glucose stimulation, we checked the phosphorylation status of 4EBP (**Figures 7C, D**). At 0.5 mM glucose, 4EBP displayed a single band (α, see **Figure 7D**) in western blot that is also visible using an anti phospho-4EBP antibody showing that 4EBP is at least partly phosphorylated. The P-4EBP/4EBP ratio increased upon glucose shift, and new 4EBP isoforms are visible (bands β and γ, see profiles in **Figure 7D**), corresponding to hyperphosphorylated 4EBP. We concluded that increase of glucose concentration induced strong phosphorylation of 4EBP that is known to decrease its affinity for eIF4E, and increases Cap-dependent translation. We also observed during a proteomic study dedicated to the analysis of the late response to glucose (4h after glucose increase), that RPS6 was phosphorylated rapidly after glucose addition, confirming the activation of the mTOR pathway (13). Interestingly, TOP-containing mRNAs are known to be extremely sensitive to mTOR regulation, that provides an attractive regulation mechanism to understand the translation upregulation of mRNAs of cluster 1 and 6 (see Discussion). Furthermore, using rapamycin, a potent inhibitor of mTOR, we observed that eIF2 dephosphorylation was independent of mTOR activation (**Figure 7E**). Since we found the kinase eEF2K in cluster 4, we asked whether eEF2 phosphorylation was affected upon glucose shift. Indeed, we found a strong reduction of eEF2 phosphorylation (**Figure 7F**, and quantification in **Figure S6C**), that is known to promote its activity for translation elongation. In conclusion, from the structural features of the clusters, we focused on mTOR and eIF2α pathways that are independently regulated to modulate translation after a short glucose stimulation, and found also an activation of the elongation factor eEF2.

## Discussion

We show here that a human pancreatic β-cell line responds to glucose by a specific regulation in protein synthesis, as revealed by polysome profiling. Interestingly, this increase in translation is unrelated to the abundance of mRNAs and consequently independent from a transcriptional regulation. Indeed, from our transcriptome analysis, a 30 min glucose shift had no effect on mRNA abundance, apart for 16 transcripts that are weakly affected. This result agrees with a microarray study performed in EndoC-βH1 in which only few genes had their mRNA levels affected after 8-hour of glucose stimulation (6).

This context where translational regulation is the major determinant of gene expression prompted us to perform the first translatome study of a human pancreatic β-cell line in response to glucose.

Following a 30 min incubation in high-glucose media, we found changes in distribution of 402 mRNAs in different ribosomal fractions. We report that two important genes involved in the regulation of secretory granules, *CHGA* and *PTPRN* are translationally co-regulated in human pancreatic β-cells. The PTPRN translation increase was also reported in mouse models (29). PTPRN belongs to the receptor protein tyrosine phosphatase (RPTP) family and regulate basal and glucose-induced insulin secretion in the mouse MIN6 cell line by increasing, presumably through stabilization, the number of insulin-containing dense core vesicles (42). Conversely, we found that the association of CHGA with heavy polysomes decreases and consequently its translation is reduced. CHGA is a member of the granin glycoprotein family, and its main intracellular function is to sort proteins into the secretory granules. CHGA is also secreted and generate several cleaved products among which pancreastatin that have been shown to act in an autocrine and paracrine fashion by inhibiting glucose stimulated insulin secretion (43).

We observed, by clustering analysis, that the identified differentially translated mRNAs could be divided into six groups based on the different changes of their mRNA levels in the three ribosomal sequenced fractions. We thus performed mRNA features analysis to identify possible mRNA features that could explain the observed different behaviours.

It is interesting to note that transcripts from cluster 4, that are translationally repressed upon glucose shift, possess a longer 3’UTR that could contain more regulatory elements, such as miRNA binding sites. Upregulation of miRNAs have been described in the presence of high glucose (44), thus, it would be interesting to analyze the modifications in miRNA activity induced by glucose in our human cellular model.

We found mRNAs from clusters 4 and 5 better translated in low glucose where eIF2 is hyperphosphorylated and, less translated in high glucose after dephosphorylation of this initiation factor. A similar situation has been observed in mice MIN6 beta cells (see introduction (11) in low glucose eIF2 phosphorylation is mediated by the Integrated Stress Response (ISR) that is suppressed upon glucose increase. Indeed, we found a similar translational behaviour for ATF4, DDIT3 and c-Jun transcripts encoding proteins associated with the ISR (45): they all belong to cluster 5. Importantly, abundance of these mRNAs decreases upon glucose increase in mouse cells but their abundance does not vary in our human beta cells.

We found mRNAs reported to contain uORFs that regulate their translation in cluster 4 and 5: the cyclic AMP-dependent transcription factor (ATF4), the activating transcription factor 5 (ATF5) (46), the transcriptional regulator CHOP (47) and eEF2K (48). As ATF4 mRNA translation has been shown to be regulated by eIF2 phosphorylation status (49), we reasoned that hyperphosphorylation of eIF2α may provide a link to the upregulation of these mRNAs.

ATF4 was the most translationally downregulated gene identified (log2 FC -1.5 in heavy polysomes). Importantly ATF4 is known to be the master regulator of cellular metabolism in response to energetic stresses and depending on the intensity and length of the stress can either favour cell survival through upregulation of autophagy related genes and amino acid transporters or enhance expression of genes involved in apoptotic processes (50). ATF5 has been recently shown to play an important role in regulating pancreatic β-cells survival (51, 52). Despite this decrease in ATF4 and ATF5, a significant transcriptomic response was not observed for 30 min (this study), or for eight hours (6) of glucose stimulation. The ATF4 protein increase is classically described to activate transcription of target genes, but this response decreases with time owing to different mechanisms that counteract ATF4 function [reviewed in (53)]. The reduction in ATF4 translation would then allow the cells to return to basal levels of ATF4 without triggering a transcriptional response.

It is interesting to note that after glucose shift, translation inhibition of eEF2K may participate in translation regulation. eEF2K phosphorylates eEF2, reducing its affinity for ribosomes, resulting in inhibition of protein synthesis (54). Indeed, we observed a strong dephosphorylation of eEF2, that would promote the elongation rate of translating ribosomes, participating to the global protein synthesis increase revealed by the measure of amino-acid incorporation.

IRESs are also RNA structures conferring a translational advantage in condition where general translation is silenced. We searched in the literature if any of the translationally repressed mRNAs were reported to contain an IRES. We found that the *RRBP1* (Ribosome-binding protein 1, cluster 5) mRNA has been shown to contain an IRES in its 5’ UTR (55). RRBP1 is a membrane-bound protein found in the endoplasmic reticulum where it enhances the association of certain mRNAs (56) and plays a role in ER morphology (57). Consequently, RRBP1 may participate to the reshaping of the translatome upon glucose induction.

eIF2α has been implicated in many physiological translation regulations, being a “funnel factor” where several signals converge to regulate its phosphorylation at serine 51, which results in cap-dependent mRNA translation repression (58), as observed for the ISR. The ISR aimed to protect cells against various cellular stresses, including viral infection, oxidative stress and ER stress. Interestingly, phosphorylated eIF2α is essential to preserve ER integrity in beta cells, and if this mechanism of protection is compromised, it would contribute to the onset of Diabetic Mellitus (59), a public health concern worldwide with an increased incidence of morbidity and mortality.

The metabolism of the beta cell is also reshaped during this early response to glucose (30 mn after glucose shift) as we found in cluster 4 transcripts coding for proteins involved in regulation of metabolic processes, cell death and response to growth factors. This translational regulation is particularly important for genes implicated in tricarboxylic acid (TCA, Krebs Cycle), acyl-CoA, and thioester metabolisms. Activation of these metabolisms, that are linked by their role in glucose consumption for energy production, is expected upon translation increase, since protein synthesis is one of the most energy costly cellular processes (60). In another study done by mass spectrometry to monitor the late response to glucose (4 hours after glucose shift), we have also observed a regulation in the amount of proteins involved in TCA metabolism and glycolysis (13).

The mRNAs that showed the strongest increase in the light polysome fraction were grouped in clusters 2 and 3. Notably these clusters showed a high GC content for both the UTR regions. The GC content in the 5’ UTR could imply a strong dependency toward helicases, and a reduced initiation activity, which could explain why these mRNAs cannot load enough ribosomes to efficiently access to heavy polysomes.

We have shown that transcripts of the two most highly translated clusters have shorter coding sequences and shorter and less complex 5’UTRs compared to the rest of the clusters. These features are characteristic of a special class of mRNAs, the TOP-mRNAs (36), which are all downstream targets of the mTOR pathway (see below). This class is defined by a 5′terminal oligopyrimidine (TOP) motif that is indeed enriched in these two highly translated clusters. Also most mRNAs from cluster 1 are known as TOP-mRNAs. The remaining 14 mRNAs of cluster 1 are most probably new TOP-RNAs, as suggested by their TOP-local score distribution. Most of the known TOP-RNAs encode proteins of the translation machinery. Accordingly, gene ontology analysis revealed that cluster 1 was enriched for categories related to structural components of the ribosomes involved in ER-related translation. Amongst the 14 new putative TOP-mRNAs that we found up-regulated in these pancreatic beta cells, we found transcripts coding for proteins acting in ubiquitin binding, cell signalling and mRNA translation. 20 mRNAs from cluster 6 were also previously reported to be TOP-RNAs (**Figure 6E**). By comparing the mRNA features of the cluster 1 and 6, we noticed similar characteristics that promote translation activation, such as short and unstructured 5’UTR, but transcripts from cluster 6 have longer CDS. This feature may explain why more ribosomes are loaded on cluster 6 mRNAs (at least 4 ribosomes) for a constant initiation rate, leading to their depletion from light polysomes and an enrichment in heavy polysomes.

The TOP-RNAs have been described to be regulated by the mTOR pathway in various situations, such as changes in nutrients and other growth signals (61). Gomez and co-workers (62), studying glucose stimulation in murine MIN6 cells, concluded that translation regulation by glucose is largely independent of mTOR but mainly dependent on the availability of the ternary complex regulated by eIF2α phosphorylation status.

In our human cellular model of beta cells, both mTOR activation and eIF2 dephosphorylation participate to the increase of mRNA translational increase. It is interesting to note that the eIF2α activation is independent of the mTOR pathway since using rapamycin, a potent inhibitor of the mTOR pathway, we have still observed the dephosphorylation of eIF2. These pathways regulate mRNA translation in particular through uORF and TOP features, that are also mutually exclusive on mRNAs, meaning that these regulations occur independently.

We concluded from our results that the glucose-dependent mTOR activation has a crucial importance for the nature of the transcripts that are regulated by glucose in human cells. As a quick response to glucose increase, TOP-RNAs allow accumulation of the translation machinery to prepare the beta cells for further protein demand due to the glucose-mediated metabolism changes.

Adaptation and response to glucose of pancreatic beta cells is critical for the maintenance of normoglycemia. Its deregulation is associated with Diabetes Mellitus. Mice models, animal or cell derived models, have tremendously contributed to our understanding of human biology. All too often, however, gene expression differ markedly from human cellular models (63). Despite extensive research in rodent models of beta cells, gene expression regulation in response to glucose remained largely unexplored in human cells beta cells. We used a human cell line, EndoC-βH2, that share similarities with primary human beta cells: they indeed express the whole machinery to transcribe, translate, store and secrete insulin ( (12, 64)). However, EndoC-βH2 cells have their own limitations. As examples, insulin content is lower that what is observed in primary human beta cells and insulin secretion upon glucose stimulation is less efficient. This might be due, among other reasons, to the fact that EndoC-βH2 cells are grown in two dimensions and in the absence of alpha and delta cells, which is different from what is observed in primary islets. Using this human cell line of pancreatic β-cells exhibiting glucose-inducible insulin secretion (12), our results emphasize a remarkable difference in gene expression regulation in response to glucose that occurs mainly at the transcriptional level in mouse and at a translational level in human pancreatic β-cell.

We have described the first genome-wide translatome study of a human pancreatic β-cell stimulated by glucose, highlighting that the response is translational and virtually independent from changes in mRNA abundance. Through the recognition of specific mRNA features, the swift translation activation is particularly efficient to increase translation machinery components. Finally, the combined mTOR and eIF2α activation that leads to the translatome reshaping governed by specific mRNA features allows a quick and direct cellular response targeting the translational regulation. These results constitute a call for a new paradigm of gene expression regulation to better understand β-cell glucose-mediated metabolism, encouraging biologists and clinicians, whenever possible, to complement their transcriptomic studies with analysis at the translational or proteomic level.

## Data availability statement

Raw sequencing data files and processes count tables for all transcripts/genes are available on Zenodo: DOI 10.5281/zenodo.4279599 (https://doi.org/10.5281/zenodo.4279599). The RNA features extraction tool, our inhouse software for the retrieval and calculation of an array of sequence features for a given set of genes is freely accessible in the GitHub repository: https://github.com/parisepigenetics/rna_feat_ext. The R-code for all the analyses in this work is available here: https://github.com/parisepigenetics/Translatome_Bcells_glucose.

## Author contributions

MB, CB, RS and BC conceived and design the analysis, MB, CB, AZ, FN, RR, LM and BC collected the data, MB, CB, AZ, FN, RR, LM and BC performed the analysis, RS and BC supervised the analysis, MB, CB, and BC wrote the first draft of the manuscript, MB, CB, FN, RR, SASA and BC contributed to manuscript revision, SASA, RS, and BC and have acquired fundings. All authors contributed to the article and approved the submitted version.

## Funding

This work was supported by the Université Sorbonne Paris Cité (USPC) 2014 [appel à projets de recherche, 2014]. Funding for open access charge: [ANR-17-CE12-0010-01]

## Acknowledgments

We thank the staff of Genom’ic platform (Cochin Institute, Paris) for library preparations and sequencing, Julia Morales (Station Biologique de Roscoff) and Yann Audic (Institute Genetics & Development, Rennes) for helpful discussions and for providing antibody samples.

## Conflict of interest

The authors declare that the research was conducted in the absence of any commercial or financial relationships that could be construed as a potential conflict of interest.

## Publisher’s note

All claims expressed in this article are solely those of the authors and do not necessarily represent those of their affiliated organizations, or those of the publisher, the editors and the reviewers. Any product that may be evaluated in this article, or claim that may be made by its manufacturer, is not guaranteed or endorsed by the publisher.

## References

[1] Hohmeier HE Newgard CB. Cell lines derived from pancreatic islets. Mol Cell Endocrinol (2004) 228:121–8. doi: 10.1016/j.mce.2004.04.017 15541576

[2] Scharfmann R Rachdi L Ravassard P. Concise review: in search of unlimited sources of functional human pancreatic beta cells. Stem Cells Transl Med (2013) 2:61–7. doi: 10.5966/sctm.2012-0120 PMC3659747 23283495

[3] Movahedi B Gysemans C Jacobs-Tulleneers-Thevissen D Mathieu C Pipeleers D. Pancreatic duct cells in human islet cell preparations are a source of angiogenic cytokines interleukin-8 and vascular endothelial growth factor. Diabetes (2008) 57:2128–36. doi: 10.2337/db07-1705 PMC2494672 18492788

[4] Schmidt SF Madsen JGS Frafjord KØ Poulsen L la C Salö S Boergesen M. Integrative genomics outlines a biphasic glucose response and a ChREBP-RORγ axis regulating proliferation in β cells. Cell Rep (2016) 16:2359–72. doi: 10.1016/j.celrep.2016.07.063 27545881

[5] Ravassard P Hazhouz Y Pechberty S Bricout-Neveu E Armanet M Czernichow P. A genetically engineered human pancreatic beta cell line exhibiting glucose-inducible insulin secretion. J Clin Invest (2011) 121:3589–97. doi: 10.1172/JCI58447 PMC3163974 21865645

[6] Richards P Rachdi L Oshima M Marchetti P Bugliani M Armanet M. MondoA is an essential glucose-responsive transcription factor in human pancreatic β-cells. Diabetes (2018) 67:461–72. doi: 10.2337/db17-0595 29282201

[7] Shalev A Pise-Masison CA Radonovich M Hoffmann SC Hirshberg B Brady JN. Oligonucleotide microarray analysis of intact human pancreatic islets: Identification of glucose-responsive genes and a highly regulated TGFβ signaling pathway. Endocrinology (2002) 143:3695–5. doi: 10.1210/en.2002-220564 12193586

[8] Itoh N Sei T Nose K Okamoto H. Glucose stimulation of the proinsulin synthesis in isolated pancreatic islets without increasing amount of proinsulin mRNA. FEBS Lett (1978) 93:343–7. doi: 10.1016/0014-5793(78)81136-3 361435

[9] Welsh M Scherberg N Gilmore R Steiner DF. Translational control of insulin biosynthesis. evidence for regulation of elongation, initiation and signal-recognition-particle-mediated translational arrest by glucose. Biochem J (1986) 235:459–67. doi: 10.1042/bj2350459 PMC1146709 3017307

[10] Permutt MA. Effect of glucose on initiation and elongation rates in isolated rat pancreatic islets. J Biol Chem (1974) 249:2738–42. doi: 10.1016/S0021-9258(19)42691-4 4597460

[11] Greenman IC Gomez E Moore CEJ Herbert TP. Distinct glucose-dependent stress responses revealed by translational profiling in pancreatic -cells. J Endocrinol (2007) 192:179–87. doi: 10.1677/joe.1.06898 PMC1831533 17210755

[12] Scharfmann R Pechberty S Hazhouz Y von Bulow M Bricout-Neveu E Grenier-Godard M. Development of a conditionally immortalized human pancreatic beta cell line. J Clin Invest (2014) 124:2087–98. doi: 10.1172/JCI72674 PMC4001549 24667639

[13] Zakaria A Berthault C Cosson B Jung V Guerrera IC Rachdi L. Glucose treatment of human pancreatic β-cells enhances translation of mRNAs involved in energetics and insulin secretion. J Biol Chem (2021) 297:100839. doi: 10.1016/j.jbc.2021.100839 34051232 PMC8253965

[14] Gosselin P Martineau Y Morales J Czjzek M Glippa V Gauffeny I. Tracking a refined eIF4E-binding motif reveals Angel1 as a new partner of eIF4E. Nucleic Acids Res (2013) 41:7783–92. doi: 10.1093/nar/gkt569 PMC3763552 23814182

[15] Ritchie ME Phipson B Wu D Hu Y Law CW Shi W. Limma powers differential expression analyses for RNA-sequencing and microarray studies. Nucleic Acids Res (2015) 43:e47–7. doi: 10.1093/nar/gkv007 PMC4402510 25605792

[16] Ewels P Magnusson M Lundin S Käller M. MultiQC: Summarize analysis results for multiple tools and samples in a single report. Bioinformatics (2016) 32:3047–8. doi: 10.1093/bioinformatics/btw354 PMC5039924 27312411

[17] Dobin A Davis CA Schlesinger F Drenkow J Zaleski C Jha S. STAR: ultrafast universal RNA-seq aligner. Bioinformatics (2013) 29:15–21. doi: 10.1093/bioinformatics/bts635 23104886 PMC3530905

[18] Li B Dewey CN. RSEM: Accurate transcript quantification from RNA-seq data with or without a reference genome. BMC Bioinf (2011) 12:323. doi: 10.1186/1471-2105-12-323 PMC3163565 21816040

[19] Wagner GP Kin K Lynch VJ. Measurement of mRNA abundance using RNA-seq data: RPKM measure is inconsistent among samples. Theory Biosci (2012) 131:281–5. doi: 10.1007/s12064-012-0162-3 22872506

[20] Lorenz R Bernhart SH Höner zu Siederdissen C Tafer H Flamm C Stadler PF. ViennaRNA package 2.0. Algorithms Mol Biol (2011) 6:26. doi: 10.1186/1748-7188-6-26 22115189 PMC3319429

[21] Sharp PM Li WH. The codon adaptation index–a measure of directional synonymous codon usage bias, and its potential applications. Nucleic Acids Res (1987) 15:1281–95. doi: 10.1093/nar/15.3.1281 PMC340524 3547335

[22] Yu G Wang L-G Han Y He Q-Y. clusterProfiler: an r package for comparing biological themes among gene clusters. OMICS: A J Integr Biol (2012) 16:284–7. doi: 10.1089/omi.2011.0118 PMC3339379 22455463

[23] Yu G Wang L-G Yan G-R He Q-Y. DOSE: an R/Bioconductor package for disease ontology semantic and enrichment analysis. Bioinformatics (2015) 31:608–9. doi: 10.1093/bioinformatics/btu684 25677125

[24] Kanehisa M Sato Y Kawashima M Furumichi M Tanabe M. KEGG as a reference resource for gene and protein annotation. Nucleic Acids Res (2016) 44:D457–62. doi: 10.1093/nar/gkv1070 PMC4702792 26476454

[25] Fabregat A Korninger F Viteri G Sidiropoulos K Marin-Garcia P Ping P. Reactome graph database: Efficient access to complex pathway data. PLoS Comput Biol (2018) 14:e1005968. doi: 10.1371/journal.pcbi.1005968 29377902 PMC5805351

[26] Tsonkova VG Sand FW Wolf XA Grunnet LG Kirstine Ringgaard A Ingvorsen C. The EndoC-βH1 cell line is a valid model of human beta cells and applicable for screenings to identify novel drug target candidates. Mol Metab (2018) 8:144–57. doi: 10.1016/j.molmet.2017.12.007 PMC5985049 29307512

[27] Gandin V Sikström K Alain T Morita M McLaughlan S Larsson O. Polysome fractionation and analysis of mammalian translatomes on a genome-wide scale. J Vis Exp (2014) 17(87):51455. doi: 10.3791/51455 PMC4189431 24893926

[28] Floor SN Doudna JA. Tunable protein synthesis by transcript isoforms in human cells. eLife (2016) 5. doi: 10.7554/eLife.10921 PMC4764583 26735365

[29] Ort T Voronov S Guo J Zawalich K Froehner SC Zawalich W. Dephosphorylation of beta2-syntrophin and Ca2+/mu-calpain-mediated cleavage of ICA512 upon stimulation of insulin secretion. EMBO J (2001) 20:4013–23. doi: 10.1093/emboj/20.15.4013 PMC149140 11483505

[30] Guest PC Rhodes CJ Hutton JC. Regulation of the biosynthesis of insulin-secretory-granule proteins. Co-ordinate translational control is exerted on some, but not all, granule matrix constituents. Biochem J (1989) 257:431–7. doi: 10.1016/S0021-9258(19)42691-4 PMC1135598 2649074

[31] Alarcón C Lincoln B Rhodes CJ. The biosynthesis of the subtilisin-related proprotein convertase PC3, but no that of the PC2 convertase, is regulated by glucose in parallel to proinsulin biosynthesis in rat pancreatic islets. J Biol Chem (1993) 268:4276–80. doi: 10.1016/S0021-9258(18)53606-1 8440711

[32] Scrucca L Fop M Murphy TB Raftery AE. Mclust 5: Clustering, classification and density estimation using Gaussian finite mixture models. R J (2016) 8:289–317. doi: 10.32614/RJ-2016-021 27818791 PMC5096736

[33] Kinsella RJ Kähäri A Haider S Zamora J Proctor G Spudich G. Ensembl BioMarts: A hub for data retrieval across taxonomic space. Database (Oxford) (2011) 2011:bar030. doi: 10.1093/database/bar030 21785142 PMC3170168

[34] Pesole G Liuni S Grillo G Ippedico M Larizza A Makalowski W. UTRdb: a specialized database of 5’ and 3’ untranslated regions of eukaryotic mRNAs. Nucleic Acids Res (1999) 27:188–91. doi: 10.1093/nar/27.1.188 PMC148131 9847176

[35] Meyuhas O Kahan T. The race to decipher the top secrets of TOP mRNAs. Biochim Biophys Acta (BBA) - Gene Regul Mech (2015) 1849:801–11. doi: 10.1016/j.bbagrm.2014.08.015 25234618

[36] Thoreen CC Chantranupong L Keys HR Wang T Gray NS Sabatini DM. A unifying model for mTORC1-mediated regulation of mRNA translation. Nature (2012) 485:109–13. doi: 10.1038/nature11083 PMC3347774 22552098

[37] Markou T Marshall AK Cullingford TE Tham EL Sugden PH Clerk A. Regulation of the cardiomyocyte transcriptome vs translatome by endothelin-1 and insulin: translational regulation of 5’ terminal oligopyrimidine tract (TOP) mRNAs by insulin. BMC Genomics (2010) 11:343. doi: 10.1186/1471-2164-11-343 20509958 PMC2900265

[38] Zhang C-S Hawley SA Zong Y Li M Wang Z Gray A. Fructose-1,6-bisphosphate and aldolase mediate glucose sensing by AMPK. Nature (2017) 548:112–6. doi: 10.1038/nature23275 PMC5544942 28723898

[39] Corradetti MN Inoki K Bardeesy N DePinho RA Guan K-L. Regulation of the TSC pathway by LKB1: Evidence of a molecular link between tuberous sclerosis complex and peutz-jeghers syndrome. Genes Dev (2004) 18:1533–8. doi: 10.1101/gad.1199104 PMC443516 15231735

[40] Gwinn DM Shackelford DB Egan DF Mihaylova MM Mery A Vasquez DS. AMPK phosphorylation of raptor mediates a metabolic checkpoint. Mol Cell (2008) 30:214–26. doi: 10.1016/j.molcel.2008.03.003 PMC2674027 18439900

[41] Gosselin P Oulhen N Jam M Ronzca J Cormier P Czjzek M. The translational repressor 4E-BP called to order by eIF4E: New structural insights by SAXS. Nucleic Acids Res (2011) 39:3496–503. doi: 10.1093/nar/gkq1306 PMC3082885 21183464

[42] Harashima S-i Clark A Christie MR Notkins AL. The dense core transmembrane vesicle protein IA-2 is a regulator of vesicle number and insulin secretion. Proc Natl Acad Sci (2005) 102:8704–9. doi: 10.1073/pnas.0408887102 PMC1150807 15939893

[43] Ahren B Bertrand G Roye M Ribes G. Pancreastatin modulates glucose-stimulated insulin secretion from the perfused rat pancreas. Acta Physiologica Scandinavica (1996) 158:63–70. doi: 10.1046/j.1365-201X.1996.525291000.x 8876749

[44] Tang X Muniappan L Tang G Ozcan S. Identification of glucose-regulated miRNAs from pancreatic {beta} cells reveals a role for miR-30d in insulin transcription. RNA (2009) 15:287–93. doi: 10.1261/rna.1211209 PMC2648717 19096044

[45] Harding HP Zhang Y Zeng H Novoa I Lu PD Calfon M. An integrated stress response regulates amino acid metabolism and resistance to oxidative stress. Mol Cell (2003) 11:619–33. doi: 10.1016/s1097-2765(03)00105-9 12667446

[46] Watatani Y Ichikawa K Nakanishi N Fujimoto M Takeda H Kimura N. Stress-induced translation of ATF5 mRNA is regulated by the 5′-untranslated region. J Biol Chem (2008) 283:2543–53. doi: 10.1074/jbc.M707781200 18055463

[47] Palam LR Baird TD Wek RC. Phosphorylation of eIF2 facilitates ribosomal bypass of an inhibitory upstream ORF to enhance CHOP translation♦. J Biol Chem (2011) 286:10939–49. doi: 10.1074/jbc.M110.216093 PMC3064149 21285359

[48] Andreev DE O’Connor PB Zhdanov AV Dmitriev RI Shatsky IN Papkovsky DB. Oxygen and glucose deprivation induces widespread alterations in mRNA translation within 20 minutes. Genome Biol (2015) 16(1):90. doi: 10.1186/s13059-015-0651-z 25943107 PMC4419486

[49] Lu PD Harding HP Ron D. Translation reinitiation at alternative open reading frames regulates gene expression in an integrated stress response. J Cell Biol (2004) 167:27–33. doi: 10.1083/jcb.200408003 15479734 PMC2172506

[50] Lindqvist LM Tandoc K Topisirovic I Furic L. Cross-talk between protein synthesis, energy metabolism and autophagy in cancer. Curr Opin Genet Dev (2018) 48:104–11. doi: 10.1016/j.gde.2017.11.003 PMC5869074 29179096

[51] Juliana CA Yang J Rozo AV Good A Groff DN Wang S-Z. ATF5 regulates β-cell survival during stress. Proc Natl Acad Sci (2017) 114:1341–6. doi: 10.1073/pnas.1620705114 PMC5307469 28115692

[52] Juliana CA Yang J Cannon CE Good AL Haemmerle MW Stoffers DA. A PDX1-ATF transcriptional complex governs β cell survival during stress. Mol Metab (2018) 17:39 -48. doi: 10.1016/j.molmet.2018.07.007 PMC6197747 30174228

[53] Kilberg MS Shan J Su N. ATF4-dependent transcription mediates signaling of amino acid limitation. Trends Endocrinol Metab (2009) 20:436–43. doi: 10.1016/j.tem.2009.05.008 PMC3587693 19800252

[54] Ryazanov AG Spirin AS. Phosphorylation of elongation factor 2: a key mechanism regulating gene expression in vertebrates. New Biol (1990) 2:843–50. 1964087

[55] Gao W Li Q Zhu R Jin J. La autoantigen induces ribosome binding protein 1 (RRBP1) expression through internal ribosome entry site (IRES)-mediated translation during cellular stress condition. Int J Mol Sci (2016) 17:1174. doi: 10.3390/ijms17071174 PMC4964545 27447629

[56] Cui XA Zhang H Palazzo AF. p180 promotes the ribosome-independent localization of a subset of mRNA to the endoplasmic reticulum. PLoS Biol (2012) 10:e1001336. doi: 10.1371/journal.pbio.1001336 22679391 PMC3362647

[57] Shibata Y Shemesh T Prinz WA Palazzo AF Kozlov MM Rapoport TA. Mechanisms determining the morphology of the peripheral ER. Cell (2010) 143:774–88. doi: 10.1016/j.cell.2010.11.007 PMC3008339 21111237

[58] Harding HP Zhang Y Ron D. Protein translation and folding are coupled by an endoplasmic-reticulum-resident kinase. Nature (1999) 397:271–4. doi: 10.1038/16729 9930704

[59] Scheuner D Mierde DV Song B Flamez D Creemers JWM Tsukamoto K. Control of mRNA translation preserves endoplasmic reticulum function in beta cells and maintains glucose homeostasis. Nat Med (2005) 11:757–64. doi: 10.1038/nm1259 15980866

[60] Buttgereit F Brand MD. A hierarchy of ATP-consuming processes in mammalian cells. Biochem J (1995) 312(Pt 1):163–7. doi: 10.1042/bj3120163 PMC1136240 7492307

[61] Laplante M Sabatini DM. mTOR signaling in growth control and disease. Cell (2012) 149:274–93. doi: 10.1016/j.cell.2012.03.017 PMC3331679 22500797

[62] Gomez E Powell ML Greenman IC Herbert TP. Glucose-stimulated protein synthesis in pancreatic β-cells parallels an increase in the availability of the translational ternary complex (eIF2-GTP·Met-tRNAi) and the dephosphorylation of eIF2α. J Biol Chem (2004) 279:53937–46. doi: 10.1074/jbc.M408682200 15475356

[63] Lin S Lin Y Nery JR Urich MA Breschi A Davis CA. Comparison of the transcriptional landscapes between human and mouse tissues. Proc Natl Acad Sci USA (2014) 111:17224–9. doi: 10.1073/pnas.1413624111 PMC4260565 25413365

[64] Scharfmann R Staels W Albagli O. The supply chain of human pancreatic β cell lines. J Clin Invest (2019) 129:3511–20. doi: 10.1172/JCI129484 PMC6715382 31478912

