# Supplementary material for: Glucose controls co-translation of structurally related mRNAs via the mTOR and eIF2 pathways in human pancreatic beta cells
Source: Front Endocrinol (Lausanne). 2022 Aug 5;13:949097. doi: 10.3389/fendo.2022.949097 (PMC9388909; doi:10.3389/fendo.2022.949097)
Supplement: Supplementary file 1 [file DataSheet_1.zip › Bulfoni_supp_fig.pdf]

Supp. Fig. 1 - Bulfoni et al.

A

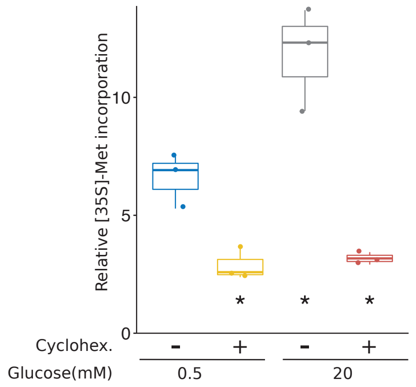

B

TranscriptID	GeneName	logFC	Adj.P.Value	Average expression
ENSG00000278272	*HIST1H3C*	0.7	7.4E-12	61.6
ENSG00000183598	***HIST2H3D***	0.8	1.1E-05	35.5
ENSG00000124575	***HIST1H1D***	0.6	2.3E-04	9.1
ENSG00000278463	*HIST1H2AB*	0.6	4.7E-04	13.1
ENSG00000177191	*B3GNT8*	0.5	5.2E-03	3.2
ENSG00000198535	*C2CD4A*	0.5	1.0E-02	1.4
ENSG00000276903	***HIST1H2AL***	0.6	1.1E-02	8.7
ENSG00000273703	*HIST1H2BM*	0.6	1.1E-02	6.6
ENSG00000185101	*ANO9*	0.6	2.1E-02	1.1
ENSG00000183248	***PRR36***	0.7	1.9E-02	0.7
ENSG00000140044	*JDP2*	0.6	2.1E-02	1.5
ENSG00000103047	*TANGO6*	0.6	3.1E-02	1.2
ENSG00000275379	*HIST1H3I*	0.5	3.2E-02	6.2
ENSG00000084628	*NKAIN1*	0.6	3.8E-02	1.0
ENSG00000007545	*CRAMP1*	0.5	4.7E-02	1.0
ENSG00000121281	*ADCY7*	0.7	3.8E-02	0.5

C

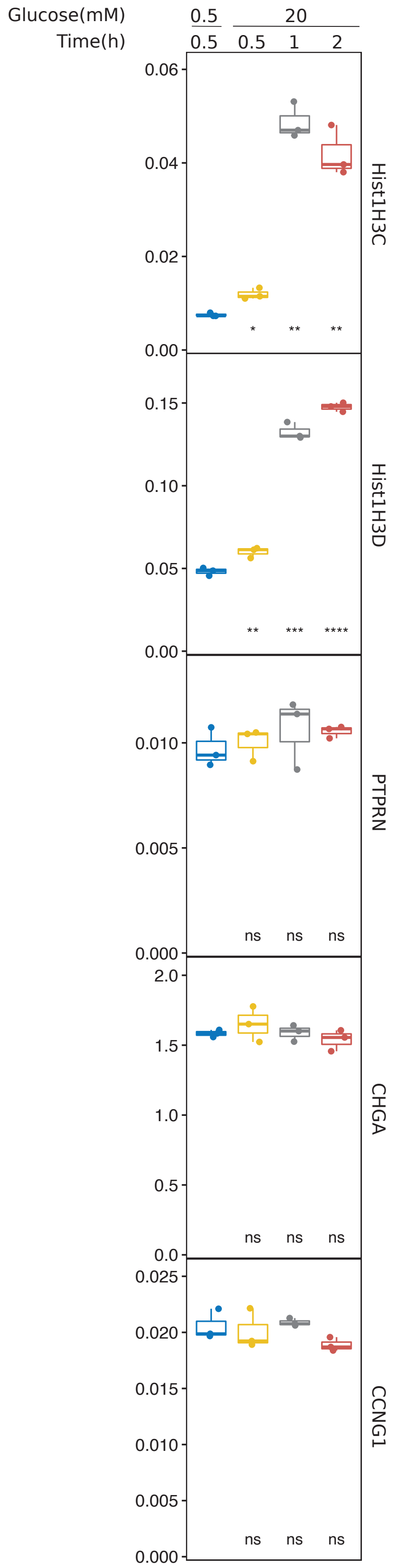

D

RNA-seq (total)	Raw Reads	Uniquely mapped
L1	19250648	79.24%
L2	16040055	78.81%
L3	16714523	77.56%
H1	17761778	78.71%
H2	17527843	79.24%
H3	18272664	78.40%

E

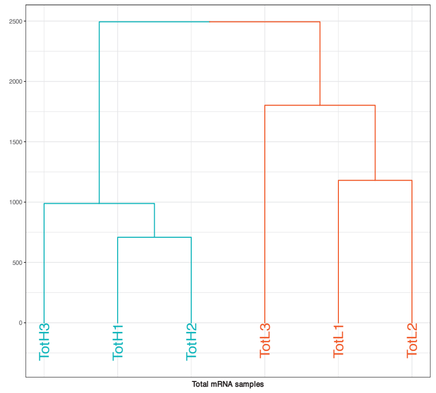

Supplementary Figure 1. A. Translation activity increase following glucose shift observed by [35S]-Methionine incorporation. Beta-cells grown in 0.5 mM glucose were shifted to 20 mM glucose during 30 min before adding [35S]-L-methionine (ref NEG709A, Perkin Elmer, final concentration 40 microCurie/ml) during 10 min. For each sample, [35S]-L-methionine incorporation into proteins was measured on duplicate aliquots after 10% TCA precipitation on Whatman as described in Costache et al., 2012 (PMID: 22425618). When indicated, cells pretreated with cycloheximide (Cyclohex.) that prevent protein synthesis were used as control. Numerical values of triplicates were represented as boxplots with pairwise comparison against condition 0.5mM without Cyclohex.(*, $p < 0.05$; Student's t test). B. Table presenting the abundance of the 16 transcripts upregulated in high glucose condition. Transcripts also upregulated in the translatome are indicated in bold. C. RT-qPCR analysis of total RNA extracts using cyclophilin as reference gene. Delta Ct values of triplicates are represented as boxplots with pairwise comparison against condition 0.5mM glucose (Student's t test; ns, $p>0.05$; *, $p<0.05$; **, $p<0.01$, ***, $p< 0.001$; ****, $p<0.0001$). D. Table summarizing the transcriptome data of total cellular extracts, after incubation of cells with low glucose (0.5 mM, L1 to L3) or high glucose (20 mM, H1 to H3). E. Hierarchical clustering of the replicates shows clear separation between low and high glucose samples.

A

Monosomes	Raw_reads	Uniquely mapped
Low_glucose rep1	10814918	61.24%
Low_glucose rep2	10578664	56.84%
Low_glucose rep3	13326344	50.12%
High_glucose rep1	12416163	60.37%
High_glucose rep2	10948574	58.57%
High_glucose rep3	10855263	64.97%
**Light Poly**		
Low_glucose rep1	9745454	75.83%
Low_glucose rep2	13997704	76.06%
Low_glucose rep3	11453671	68.52%
High_glucose rep1	10723806	73.56%
High_glucose rep2	11572776	69.51%
High_glucose rep3	8762845	68.10%
**Heavy Poly**		
Low_glucose rep1	12051978	74.58%
Low_glucose rep2	9749807	74.69%
Low_glucose rep3	7871513	73.96%
High_glucose rep1	8476376	73.78%
High_glucose rep2	8985906	80.24%
High_glucose rep3	13196915	77.64%

B

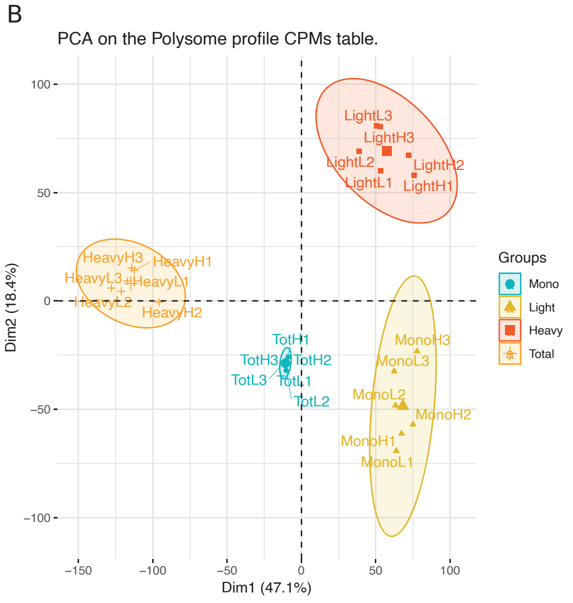

C D

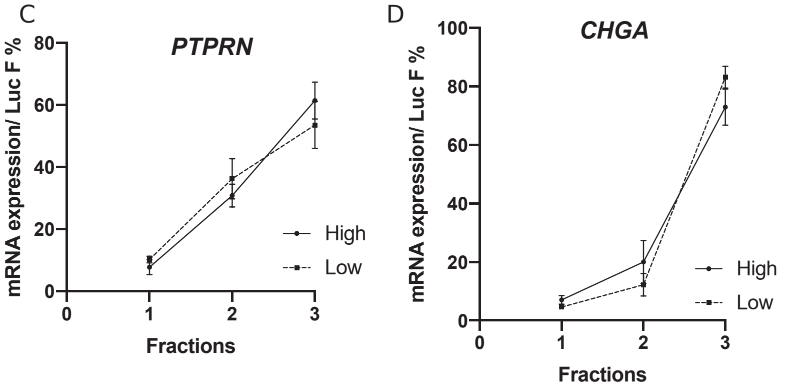

E

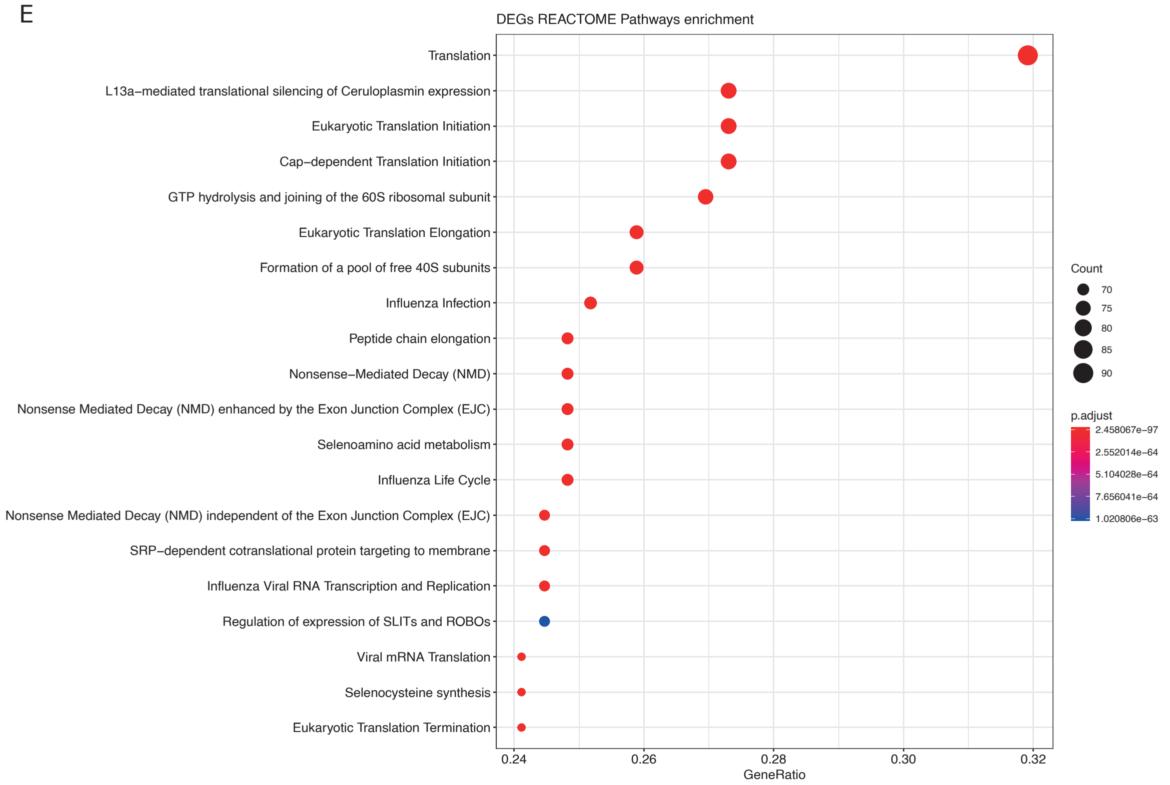

Supplementary Figure 2. A. Table summarizing the number of raw reads and the percentage of uniquely mapped reads for each replicate in each condition. B. PCA analysis of aligned reads from each pool of fractions of the polysome profiling. C-D. RT-qPCR analysis on each of the pool of fractions. E. Dot-plot of the top 20 most significant REACTOME pathways enriched in the differentially translated genes.

Supp. Fig. 3 - Bulfoni et al.

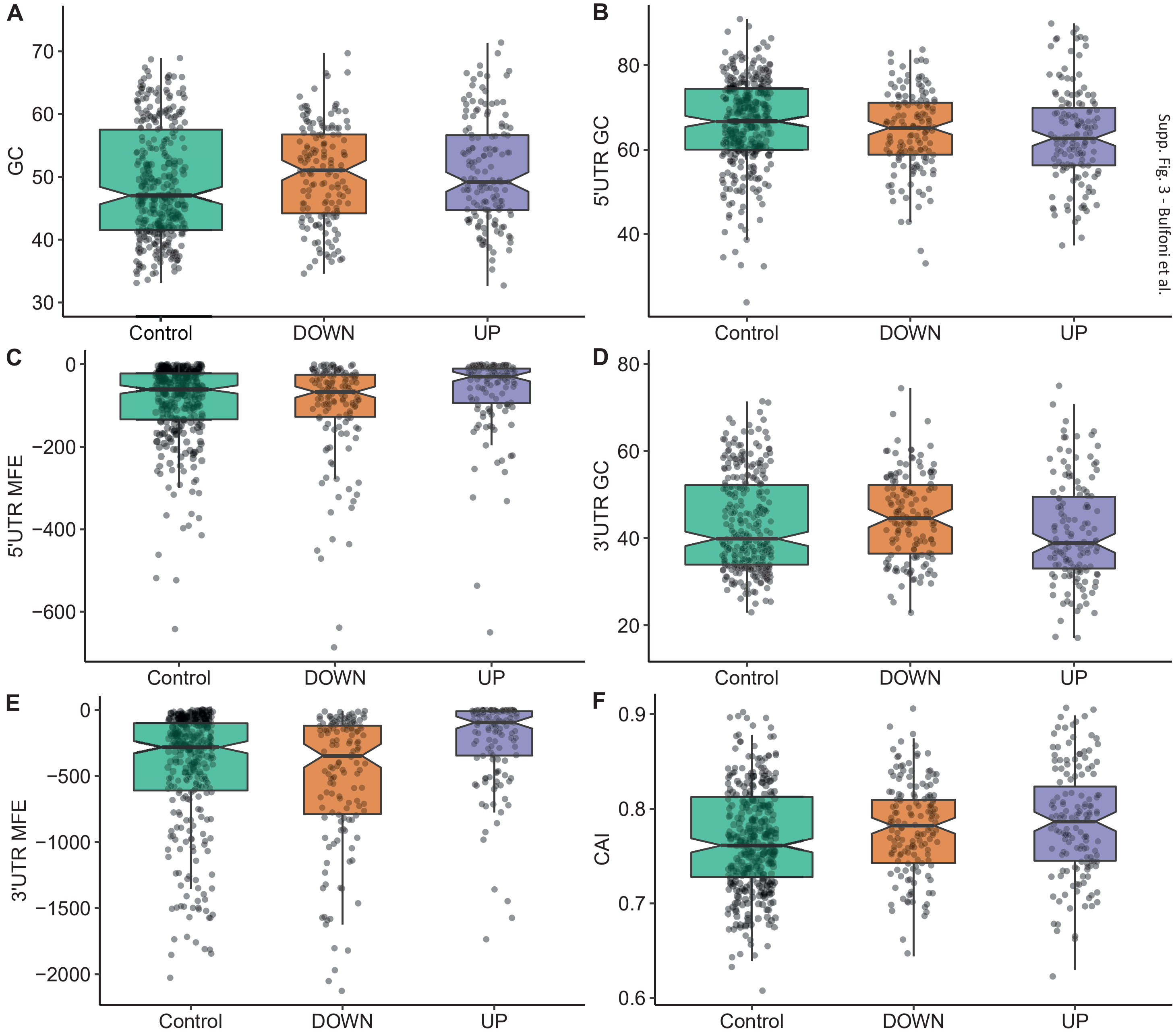

Supplementary Figure 3. mRNA features analyses for 3 groups of mRNAs based on the translation ratio between high and low glucose (see Figure 3)).

Supp. Fig.4 - Bulfoni et al.

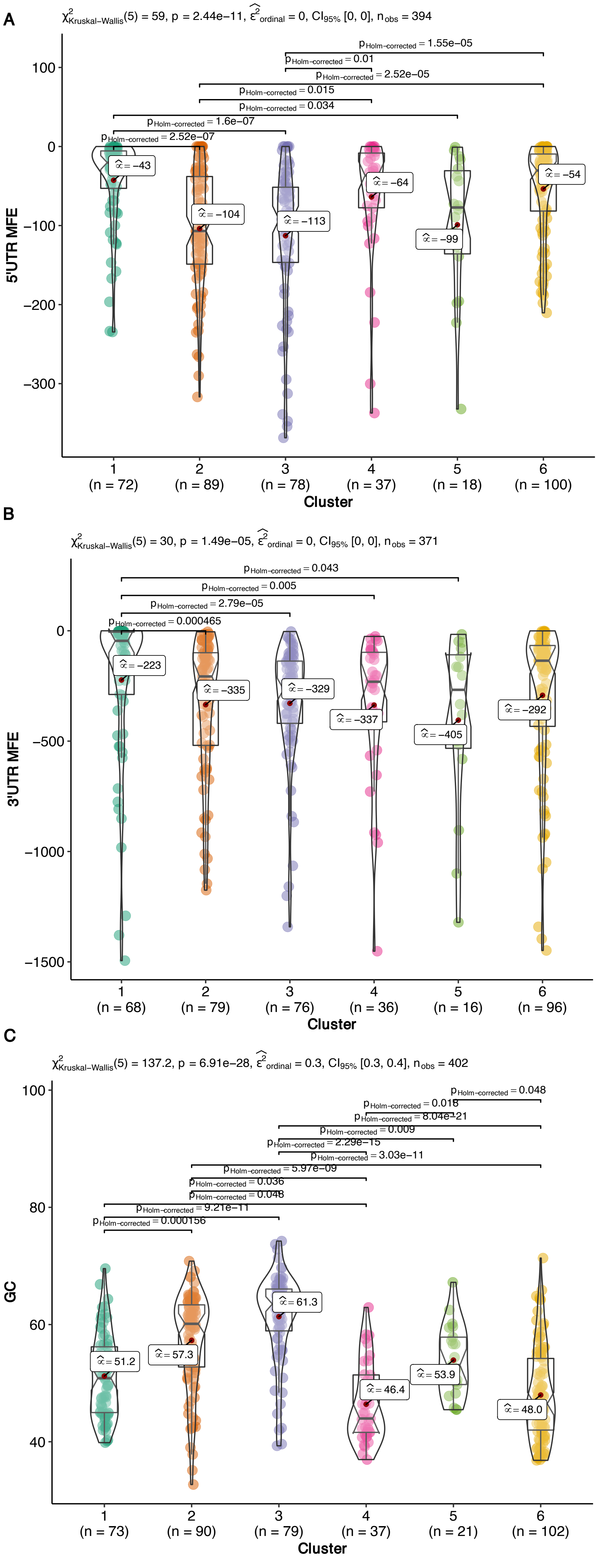

Supplementary Figure 4 : Boxplots and statistical analyses for mRNA features. Each plot corresponds to an analysis of a particular feature for all the 6 clusters of translation behaviour. The Kruskal-Wallis H-test is used for all the group comparisons to test for median differences with the Dunn test for each pairwise comparison. The test statistics are shown in the subtitle and each significant pairwise comparison (corrected for multiple testing) is plotted only for significant pairwise differences.

Supp. Fig. 5 - Bulfoni et al.

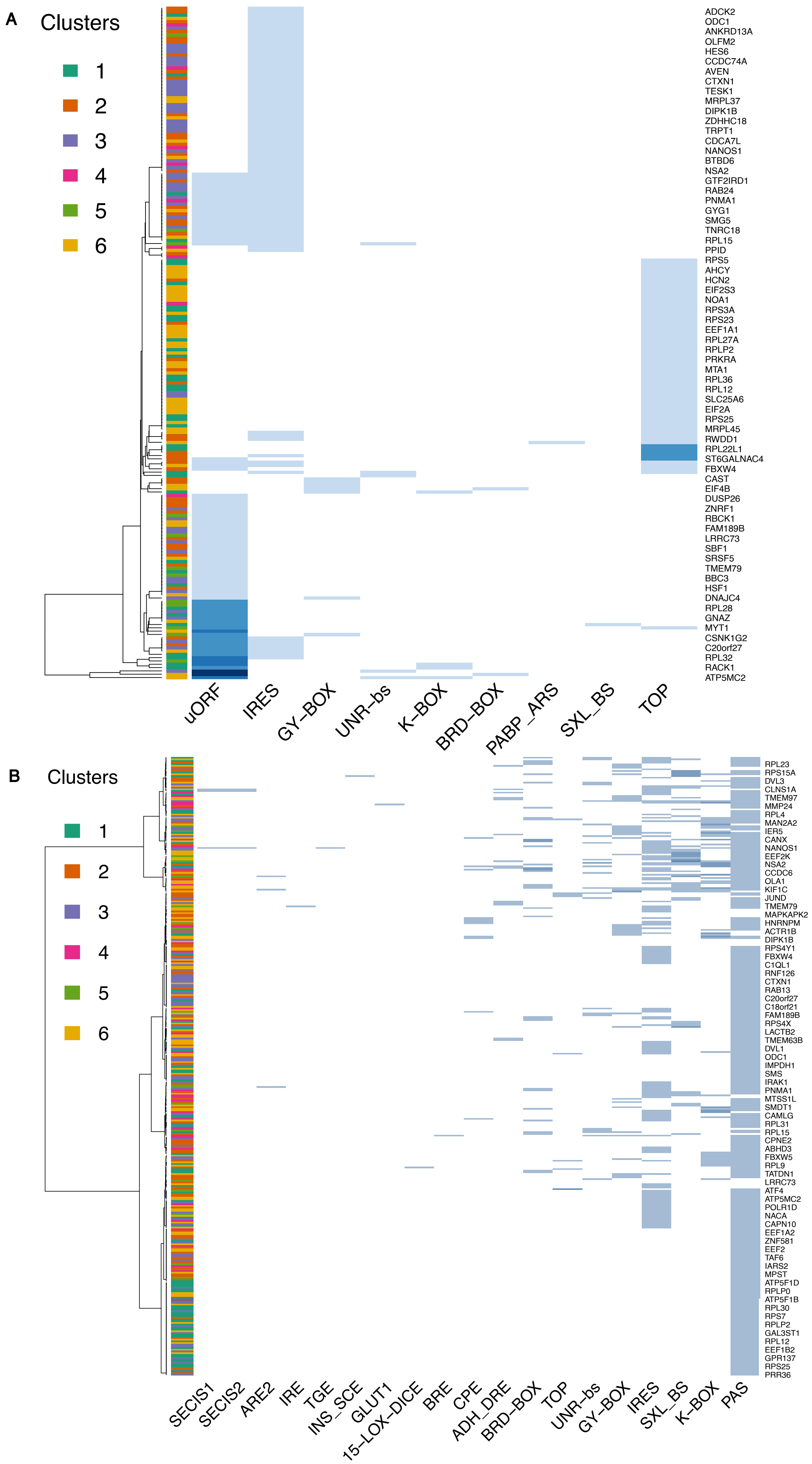

Supplementary Figure 5. Functional motifs analysis of the 5'UTRs (A) and 3'UTRs (B) by interrogating the UTRdb. Ensembl IDs of transcripts are indicated on the right side, ordering of the transcripts was done by clustering of features.

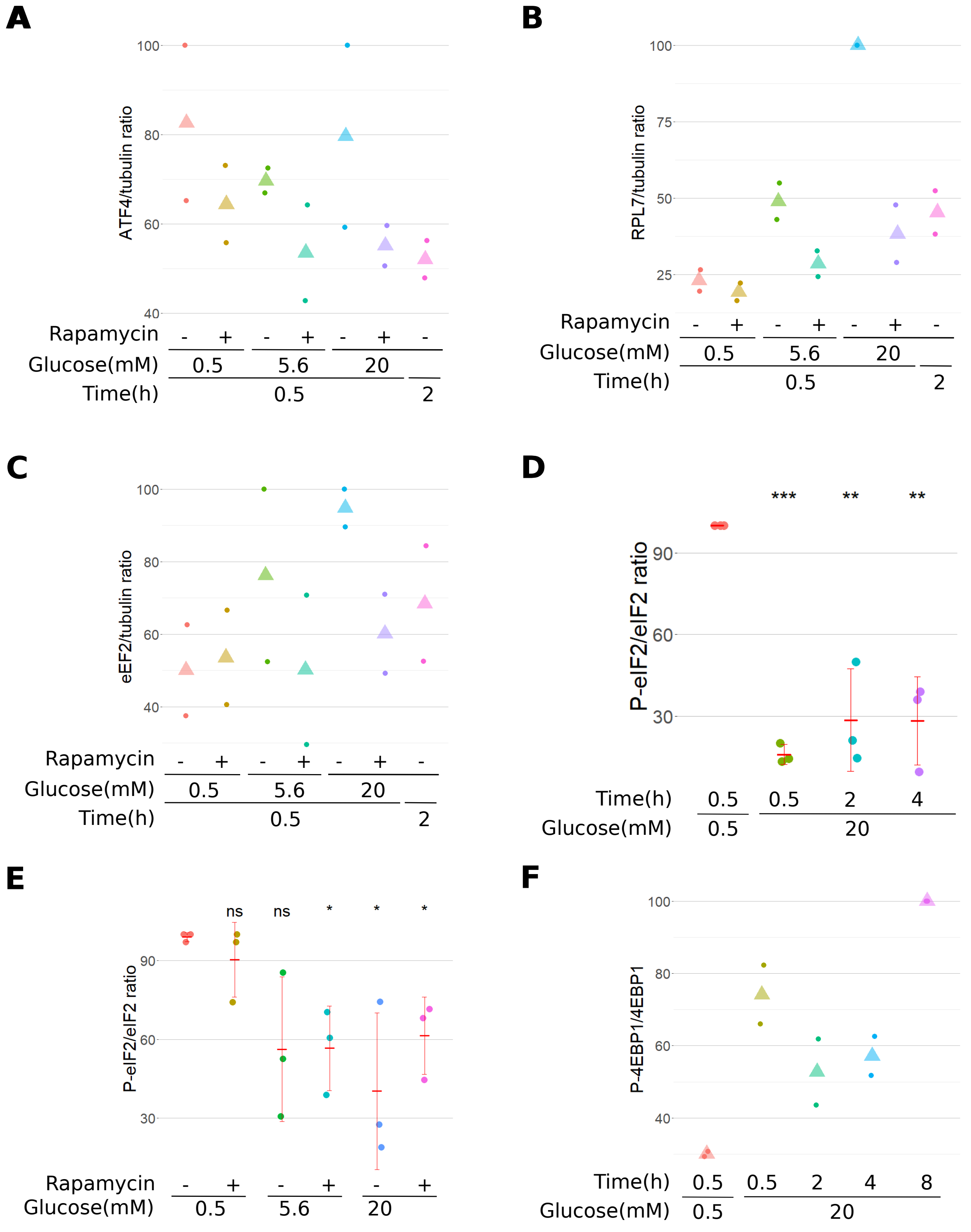

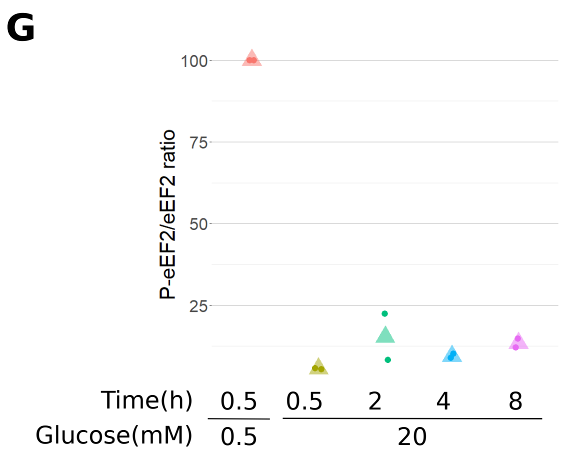

Supplementary Fig. 6 : Quantification of the Western Blots of Fig. 7 : experiments were repeated at least two times, D (data from Fig. 7B), E (from Fig. 7E), numerical values of triplicates from one experiment are shown along with the mean (horizontal red dash) +/- SD (vertical red line) with pairwise comparison against condition 0.5 mM (Student's t test; *, $p<0.05$, **, $p< 0.001$, ***, $p<0.0001$). A-C (from Fig. 7A), F (from Fig. 7C), G (from Fig. 7F): numerical values of duplicates from one experiment are shown along with the mean (triangles). Tubulin was used as loading control for normalization.
